# Nanoscaled Discovery of a Shunt Rifamycin from *Salinispora arenicola* Using a Three-Color GFP-Tagged *Staphylococcus aureus* Macrophage Infection Assay

**DOI:** 10.1021/acsinfecdis.3c00049

**Published:** 2023-07-11

**Authors:** Nhan T. Pham, Joana Alves, Fiona A. Sargison, Reiko Cullum, Jan Wildenhain, William Fenical, Mark S. Butler, David A. Mead, Brendan M. Duggan, J. Ross Fitzgerald, James J. La Clair, Manfred Auer

**Affiliations:** † School of Biological Sciences, The University of Edinburgh, The King’s Buildings, Edinburgh EH9 3BF, U.K.; ‡ The Roslin Institute, The University of Edinburgh, Easter Bush Campus, Midlothian EH25 9RG, U.K.; § Center for Marine Biotechnology and Biomedicine, Scripps Institution of Oceanography, University of California at San Diego, La Jolla, California 92093-0204, United States; ∥ Exscientia Oxford Science Park, The Schrödinger Building, Oxford Science Park, Oxford OX4 4GE, U.K.; ⊥⊥ Xenobe Research Institute, P. O. Box 3052, San Diego, California 92163, United States; # Terra Bioforge Inc., 3220 Deming Way Suite 100, Middleton, Wisconsin 53562, United States; ∇ Skaggs School of Pharmacy and Pharmaceutical Sciences, University of California, San Diego, 9500 Gilman Drive, La Jolla, California 92093, United States; ○ Department of Chemistry and Biochemistry, University of California at San Diego, La Jolla, California 92093-0358, United States

**Keywords:** fluorescence imaging assay, macrophage, rifamycin, *Salinispora arenicola*, *Staphylococcus aureus*

## Abstract

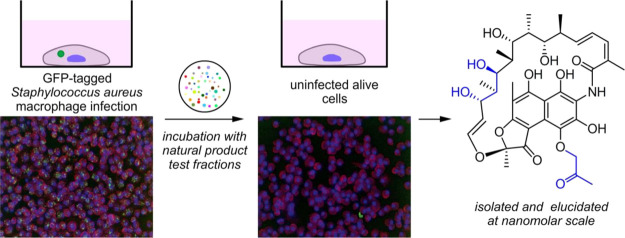

Antimicrobial resistance has emerged as a global public health threat, and development of novel therapeutics for treating infections caused by multi-drug resistant bacteria is urgent. *Staphylococcus aureus* is a major human and animal pathogen, responsible for high levels of morbidity and mortality worldwide. The intracellular survival of *S. aureus* in macrophages contributes to immune evasion, dissemination, and resilience to antibiotic treatment. Here, we present a confocal fluorescence imaging assay for monitoring macrophage infection by green fluorescent protein (GFP)-tagged *S. aureus* as a front-line tool to identify antibiotic leads. The assay was employed in combination with nanoscaled chemical analyses to facilitate the discovery of a new, active rifamycin analogue. Our findings indicate a promising new approach for the identification of antimicrobial compounds with macrophage intracellular activity. The antibiotic identified here may represent a useful addition to our armory in tackling the silent pandemic of antimicrobial resistance.

Traditional targeted high-throughput screening (HTS) of large chemical libraries created by combinatorial chemistry has proved very efficient in finding hits and lead compounds for a variety of indications. It was thought that this process would be similarly successful in discovering antibiotics. However, several attempts to use HTS against large corporate combinatorial libraries have failed to identify new broad-spectrum antibiotics.^1−3^ Furthermore, common antimicrobial screening methods that analyze bacterial growth via target-based biochemical assays can overlook possible cytotoxic effects and permeability to cellular membranes.^4^ For these reasons, allied to the automation and development of high-content screening (HCS) technologies, drug discovery strategies have turned to cell-based assays that employ the microbe in its cellular environment.^5^ In 2009, Christophe et al. employed HCS to identify synthetic chemical compounds to inhibit the intracellular replication of *Mycobacterium tuberculosis* within macrophages.^6^ Subsequent studies led to the identification of a potent clinical candidate, telacebec (Q203), for the treatment of tuberculosis (Phase II study completed) as well as for Buruli-ulcer (Phase I study underway in Korea).^7^ Related assays have been used to identify antimicrobial compounds against *Salmonella typhimurium*, *Plasmodium falciparum*, and *Leishmania* spp.^8,9^ These assays are especially important for the discovery of antimicrobial agents active against intracellular pathogens or pathogens that survive and replicate inside host cells during their infection cycle.^5^

*Staphylococcus aureus* is a leading cause of hospital and community acquired infections, and its ability to develop resistance to antimicrobial agents makes it a priority for the development of agents with new modes of action, superior toxicity profiles, and intravenous (IV)/oral switch administration. During *S. aureus* pathogenesis, the interaction with host macrophages has a pivotal role in determining the outcome of infection. *S. aureus* has evolved multiple strategies to survive within, manipulate, and escape from macrophages.^10^ The macrophage intracellular environment not only shields the bacteria from most antimicrobials but also can support the development of persister cells with an antimicrobial tolerant phenotype.^11,12^ To address these issues, we developed a high-content human macrophage infection assay (Figure 1a) to identify new antibiotics from marine microbial test fractions with antimicrobial properties against multi-drug-resistant *S. aureus*. With this assay, we identified intracellular *S. aureus* growth inhibitors in macrophages.^10^

**Figure 1:**
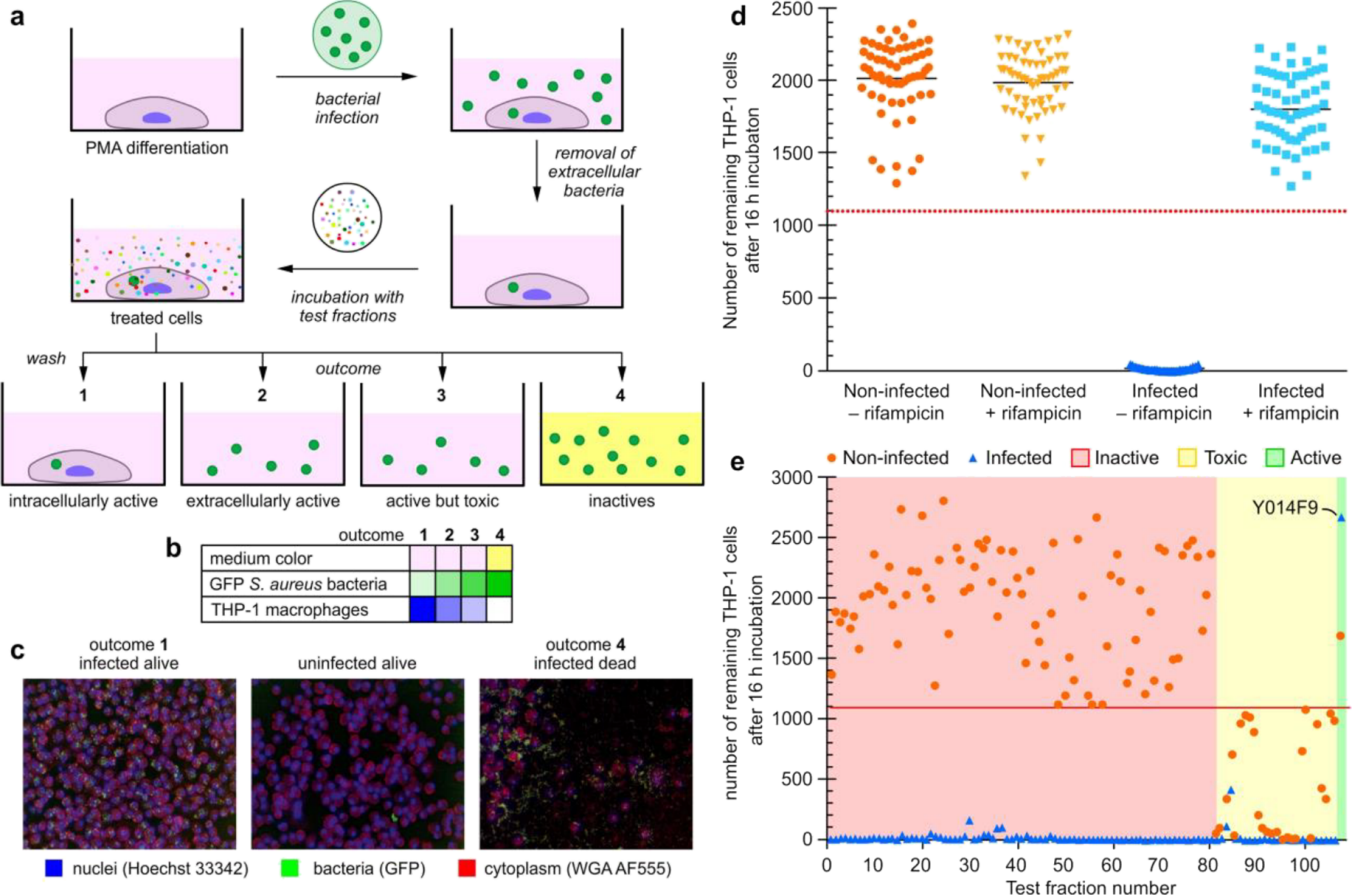
Fluorescent macrophage infection assay. (a) Schematic representation of the three-color confocal fluorescence macrophage infection assay run in 96-well plate format on a PerkinElmer Opera instrument using a 20× objective. The assay process begins with infecting PMA differentiated THP-1-macrophage cells with GFP-USA300 *S. aureus*. Co-culturing of these cells results in infected mammalian cells after washing and removal of the media. The resulting bacterial infected cells are then subjected to test samples, which can result in four different outcomes. The first, arising from intracellular active compounds, is identified by a significant number of infected THP-1 cells remaining after the overnight incubation (indicated as dark blue in Figure 1b), and the cell media is red/pink (indicated as rose in panel b). The number of GFP-USA300 *S. aureus* bacteria is low (indicated as light green in panel b). The next two, extracellular active and active but toxic compounds, are marked by a low number of remaining THP-1 cells (indicated as light blue in panel b), some extracellular bacteria (indicated as lighter green in Figure 1b, and red/pink cell media (indicated as rose in panel b). The only difference between these two outcomes is that in the non-infected control, only a few to no THP-1 cells are found for toxic compounds. The final outcome, arising from non-active compounds, contains an absence of THP-1 cells as well as yellow cell media (panel b, lane 4), caused by saturated growth of bacteria (indicated by a dark green colored field in panel b, lane 4), which turns the cell media acidic, causing a color change to yellow. The cell membranes and the nuclei of the THP-1 macrophages were stained with wheat germ agglutinin Alexa Fluor 555 (WGA AF555) and Hoechst 33342 to enable blue/green/orange detection scheme. (b) Heatmap showing the outcome for each of the four different possibilities. The color in the table illustrates whether there is a color change in the cell medium, and the strength of the color illustrates the number of bacteria or THP-1 cells found in the sample. Medium color: color change from pink (red) to yellow only for outcome 4. GFP *S. aureus* bacteria (green) are most abundant for outcome 4. THP-1 macrophages (blue) are most abundant for outcome 1 and absent for outcome 4. (c) Typical Opera images for the various outcomes of the assay, THP-1 nuclei and plasma membrane are blue and red, respectively, and *S. aureus* bacteria are in green. Left image visualizes the outcome for an intracellularly active compound, the middle for an extracellularly active compound, and right image for an inactive compound. (d) Assay sensitivity. The separation between positive control containing infected THP-1 cells in 50 nM rifampicin (light blue square) and negative control (dark blue upright triangle) containing infected THP-1 cells in media only. For comparison, non-infected THP-1 cells with (yellow upside-down triangle) and without (orange round dots) 50 nM rifampicin are also shown. The red dashed line is the hit threshold which is the lower 3σ (standard deviation) or limit of the average of infected THP-1 cells in 50 nM rifampicin (parameters providing in the Supporting Information). (e) Results of the screen of 108 marine microbial test fractions. More than 80 fractions were inactive (red shaded area), and the rest of the fractions were toxic to THP-1 cells (yellow shaded area) except fractionY014F9, which was identified to be active, with a high number of infected and non-infected THP-1 cells remaining after overnight incubation.

## Results

### Design and Implementation of a Three-Color Green Fluorescent Protein (GFP)-Tagged *S. aureus* Macrophage Infection Assay

To address the failure of conventional screening methods to identify new antibiotics from drug-like small molecule chemical libraries, we developed a new *S. aureus* macrophage infection assay. Using this assay, we screened 108 marine natural product fractions, consisting of ∼1080 compounds (average of 10 compounds per fraction), for the ability to inhibit intracellular *S. aureus* growth and survival inside macrophages. Our assay (Figure 1a) utilized THP-1-derived macrophages infected with a community associated, GFP-expressing methicillin resistant strain of *S. aureus* (MRSA) (USA300 LAC-GFP).^13^ THP-1 cells are human monocytic cells that are differentiated into macrophages using phorbol myristate acetate (PMA). These cells present an inflammatory macrophage phenotype, are highly phagocytic,^14^ and have been extensively used to study bacterial-macrophage interactions^15^ and antimicrobial intracellular activity.^14,16^ Despite some differences when compared with primary cells,^17^ the use of this cell line allowed us to overcome issues with availability, donor variability, and ethical considerations associated with primary human blood monocyte-derived cultures, as well as high reagent costs, and the time consuming protocols of preparing macrophage-like cells from human embryonic stem cells.^18^ The differentiated, adherent THP-1 cells were infected with USA300 LAC-GFP *S. aureus* at a multiplicity of infection (MOI) of 10 for 1 h, and to test the intracellular activity of the compounds, the extracellular bacteria were killed by incubation with gentamicin prior to the addition of the test fractions.

Natural product fractions were added to the cells at 25 and 5 μg/mL and incubated for 16 h. Subsequently, cells were fixed with 4% paraformaldehyde (PFA) and stained with Hoechst 33342 (nuclei) and Wheat Germ Agglutinin Alexa Fluor 555 Conjugate (plasma membrane) giving a three-color read out with *S. aureus* stably expressing GFP.

This assay has four possible outcomes: inactive test fraction, active but cytotoxic, extracellular active, and intracellular active (Figure 1a–c). As in the negative control (media only), incubation with inactive compounds allowed the bacteria to kill the THP-1 macrophages, escape the intracellular environment, and multiply in the cell culture media during overnight incubation, lowering the pH of the media to change its color from red to yellow. Extracellularly active fractions show no color change of the media due to absence of both saturated bacterial growth and live THP-1 macrophages. We focused on identifying intracellular active compounds associated with no media color change and the presence of live THP-1 cells after the overnight incubation (further details in the Methods section). As shown in Figure 1d, we were able to validate our assay using rifampicin as an active model drug. Over multiple repetitions of the imaging process, infected THP-1 cell death occurred within 16 h, which was prevented in cultures treated with rifampicin (Figure 1d).

### Screening of Marine Microbial Test Fractions Leads to a Hit Fraction Y014F9

Next, we turned to chromatographically fractionated marine microbial fractions (test fractions) developed in the Fenical laboratory from a large repository of marine microbes (>17,000 strains).^19^ A panel of 108 test fractions was selected that contained ∼10 compounds per fraction at a concentration of 10–50 mg/mL in DMSO. Here, the goal was to complete the entire discovery effort with less than 1 mL of each test fraction and finish with a characterized active compound. To achieve this, we needed to determine the limits of this screening effort and began by screening 100 μL aliquots of these fractions. From each fraction, a 500 μL aliquot was saved for compound purification. Figure 1e shows the outcome of the primary screen of the 108 fractions. About a quarter of the fractions showed toxicity (at 25 μg/mL) against macrophages. This can be seen by the low number of THP-1 macrophages in the non-infected controls, indicated by the orange data points below the red line in the yellow shaded part of Figure 1e. There were no remaining infected THP-1 macrophages for all but a few fractions. Only fraction Y014F9 yielded macrophage cell numbers exceeding our hit criteria (blue data point above the red line marked with Y014F9 in Figure 1e).

### Compound Identification and Validation through Microbial Reculturing

**Figure 2:**
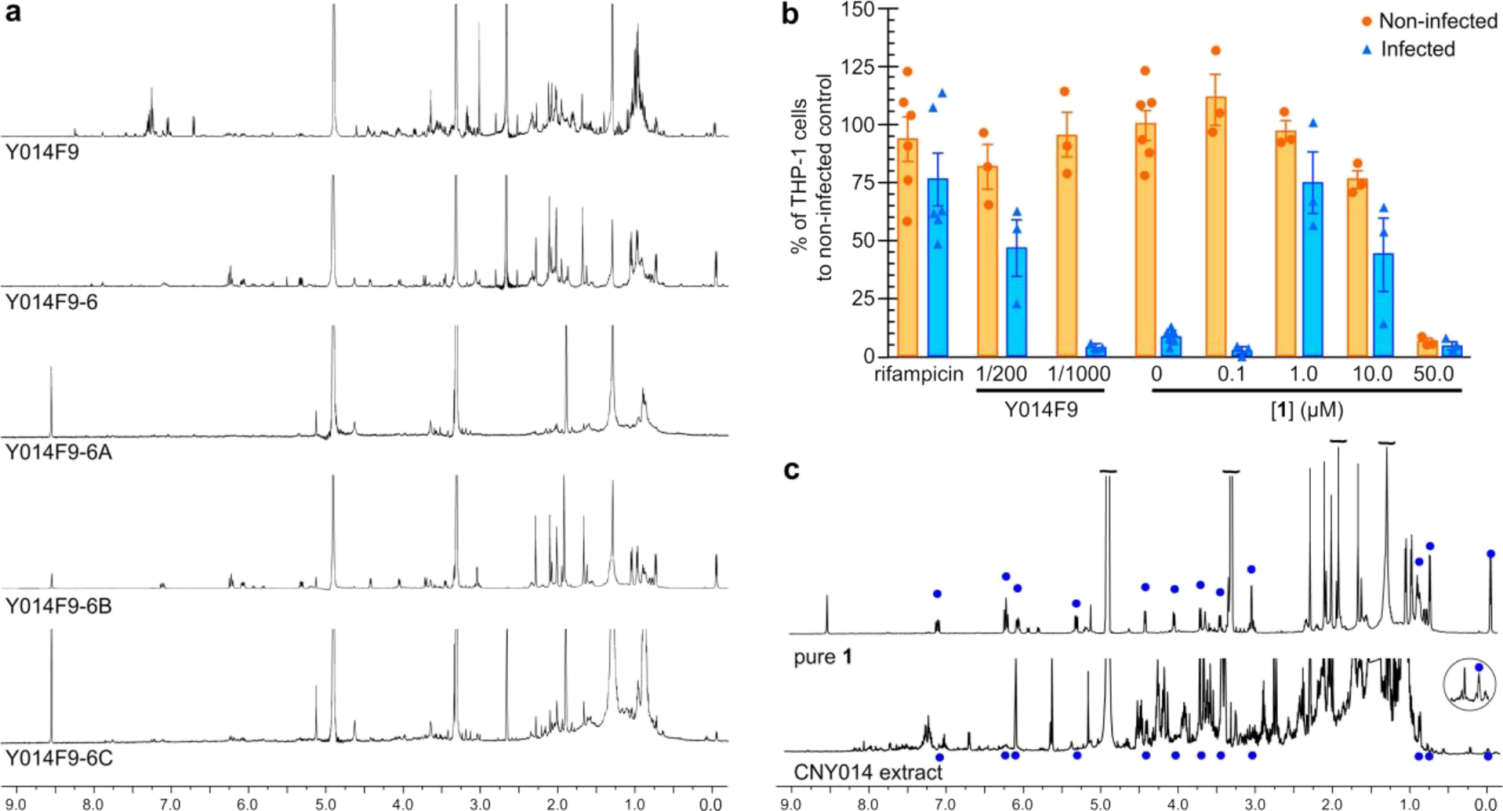
Nanomole-scaled isolation of **1** from the test fraction Y014F9. (a) ^1^H NMR spectra collected on 50 μL sample of the test fraction Y014F9. A 100 μL sample of the test fraction in DMSO was then further fractionated into eight bands by pTLC to afford fractions Y014F9-1 to Y014F9-8. The second purification of Y014F9-6 afforded three cuts Y014F9-6A to Y014F9-6C. (b) Testing of compound **1** obtained from Y014F9 at 0.1, 1.0, 10, or 50 μM in the THP-1 macrophage infection assay. For comparison, 50 nM rifampicin and the parent test fraction Y014F9 at 1/200 and 1/1000 dilution are shown. (c) ^1^H NMR spectra comparing purified **1** against the CYN014 extract.

With a hit fraction (Y014F9) identified, we turned to methods scaled at the nanomolar level (nanoscaled) to conduct the assay-guided fractionation directly from the screening aliquot. A sample of the DMSO stock of fraction Y014F9 (100 μL) was dried by airflow and redissolved in MeOH (50 μL). A 50 μL solution of the MeOH stock was separated using a 5 cm × 20 cm, pTLC plate (250 μm) (eluent 1:9 mixture of MeOH:EtOAc) into eight bands (Y014F9-1 to Y014F9-8) (Supporting Table 1). Each band was submitted to nanoscaled-NMR analyses and recollected after use. Half of the remaining material was resubmitted to the assay, while the other half was saved for further purification. Application of the assay to the fractions identified Y014F9-6 (NMR spectrum provided in Figure 2a) as the most active. Using the remaining material, we repeated the fractionation to afford three bands Y014F9-6A to Y014F9-6C (NMR spectra provided in Figure 2a). After collection of NMR data, these samples were then resubjected to the assay identifying Y014F9-6B as the most active (Figure 2b).

**Figure 3:**
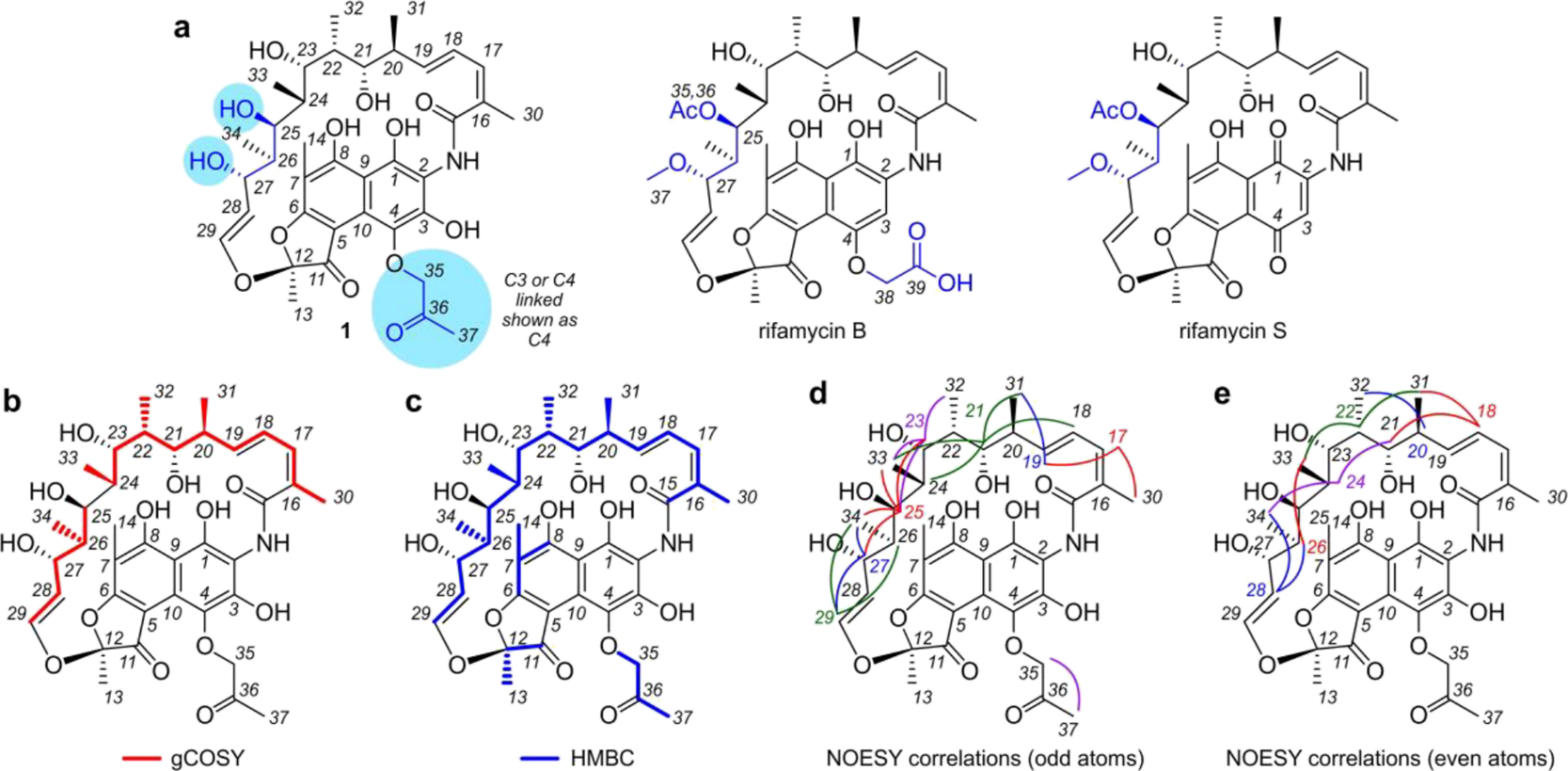
Structure elucidation. (a) Structures of 4-(propan-2-one)-25-*O*-deacetyl-27-*O*-desmethylrifamycin (**1**), rifamycin B, and rifamycin S. Reference NMR data sets were collected from rifamycin B (Supporting Table S3) and rifamycin S (Supporting Table S4) to aid in the structure elucidation process. (b) Summary of the ^1^H-^1^H gCOSY data obtained from **1** in CD_3_OD (Supporting Table S2). Key gCOSY (red) correlations are shown on **1**. (c) Summary of the ^1^H-^13^C HMBC data obtained from **1** in CD_3_OD (Supporting Table S2). Key HMBC (blue) correlations are shown mapped on **1**. (d, e) Stereochemical assignments in **1** were confirmed by tabulating the ^1^H-^1^H NOESY data from **1** (Supporting Table S2) and comparing them to that expected from X-ray crystal structural data from rifampicin (Supporting Figure 3). Key NOE interactions are displayed in (d) for the odd numbered protons from H17-H29 and (e) even numbered protons from H18–H28. Search engines including Scifinder, MarinLit, and Coconut indicate that **1** is a new member of the rifamycin class. While 25-*O*-deacetyl-27-*O*-desmethylrifamycin is a known motif, all evidence from these searches suggest that this is the first member of this large family of natural products with a propan-2-one modification.

We then recultured the microbe, *Salinispora arenicola* strain CNY-014, that had produced test fraction Y014F9. Fermenting at 5 L scale returned 400 mg of crude extract, which after NMR-guided fractionation afforded 8 mg of fraction S10 (Supporting Table 1). Further purification of this material by prep-TLC (5 cm × 20 cm, 250 μm pTLC plate and elution with 1:9 mixture of MeOH:EtOAc) afforded 520 μg (estimated by NMR) of rifamycin analogue **1** (Figure 3a). By ^1^H NMR analysis (Figure 2c), this material was identical to that obtained from purification of Y014F9-6B (Figure 2a), thereby confirming the source as well as providing sufficient material for NMR analyses. The low yield (0.13%) of **1** indicated that it was a minor component of the extract, a fact that was evident by comparing the NMR spectrum of pure **1** to its parent extract (Figure 2c).

### Structure Elucidation and Activity Validation of the Active Component

With 520 μg of **1**, we were able to obtain a structure elucidation data set, including ^1^H-^1^H gCOSY, ^1^H-^13^C HSQC, ^1^H-^13^C HMBC, and ^1^H-^1^H NOESY and HRMS spectra. HRMS analyses returned an *m*/*z* [M-H]^−^ of 712.2969, which was consistent with the formula of C_37_H_47_NO_13_ (calcd [M-H]^−^ of 712.2975). While database searching (MarinLit, SciFinder) failed to produce hits, we were rapidly able to build two fragments by interpretation of ^1^H-^1^H gCOSY and ^1^H-^13^C HSQC NMR data (red lines, Figure 3b). ^1^H-^13^C HMBC NMR data were then used to identify the missing correlation between C24 and C25 in the ^1^H-^1^H gCOSY data, resulting in a single 20 carbon fragment (blue line, Figure 3c).

The ansamycin family of polyketides^20^ has been previously reported from *Salinispora*,^21^ and resonances in the NMR spectra of **1** supported their presence. In particular, the presence of three olefinic protons coupled within a diene (confirmed by ^1^H-^1^H gCOSY and ^1^H-^13^C HMBC data), four oxygen substituted methines and four methine-attached methyl groups. Furthermore, the ^1^H-^1^H gCOSY and ^1^H-^13^C HMBC data showed additional correlations with a *trans*-disubstituted olefin at C28–C29 with a ^3^*J*_HH_ coupling constant of 12.7 Hz. Taken together, these NMR data suggested that **1** was a member of the rifamycin subclass of the ansamycin family.^22^

We then collected NMR data (^1^H-^1^H gCOSY, ^1^H-^13^C HSQC, ^1^H-^13^C HMBC, and ^1^H-^1^H NOESY) for rifamycin B (Figure 3a) and rifamycin S (Figure 3a) as tools to aid structure elucidation. First, we determined that the C28–C29 olefin in **1** contained comparable proton coupling constants (^3^*J*_27–28_ of 7.0 Hz, ^3^*J*_28–29_ of 12.7 Hz, and ^4^*J*_27–29_ of 1.4 Hz, Supporting Table 2) to those observed in rifamycin B (^3^*J*_27–28_ 7.0 Hz, ^3^*J*_28–29_ 12.7 Hz, and ^4^*J*_27–29_ 1.0 Hz, Supporting Table 3) and rifamycin S (^3^*J*_27–28_ 7.9 Hz, ^3^*J*_28–29_ 12.6 Hz, and ^4^*J*_27–29_ 0.8 Hz, Supporting Table 4), therein indicating that the olefin residue observed by ^1^H-^1^H gCOSY (Figure 3b) and ^1^H-^13^C HMBC (Figure 3c) analyses was likely a *trans*-olefin, as observed in rifamycins B and S.

Chemical shift analyses (^1^H and ^13^C, Supporting Figure 1) were used to identify the functionality in **1** that was different for that in rifamycins B or S. Overall the chemical shifts of **1** shared a strong similarity with selected shifts for rifamycin B and rifamycin S (Supporting Table 5). The aromatic proton H3 was not present (7.37 bs in rifamycin B and 7.63 s in rifamycin S, Supporting Table 5), which indicated functionalization (commonly observed as oxidation) at C3. Functional modification between C25 and C28 was suggested by chemical shift differences observed for H25, H27, C25, C27, and C28. Using these data as a guide, we were able to determine that **1** had secondary alcohols at positions C25 and C27, was not acetylated at C25 as observed in rifamycin B (C35 δ172.6, H36 δ 2.02, C26 δ 20.8, Supporting Table 3), and was not O-methylated at position C27 (H37 δ 3.03, C37 δ 57.1, Supporting Table 3). In addition to these structural differences (blue highlights, Figure 3a), the NMR data for **1** (Supporting Table 5) were not consistent with the same glycolic acid present at C4 in rifamycin B, as noted by the lack of signals seen for rifamycin B (H38 δ 4.74 and C38 δ 67.7 ppm). In contrast, **1** contained a 2-propanone functionality whose position was tentatively assigned to C4 (see note in Figure 3a). Unfortunately, complete assignment of this position was complicated by the fact that in **1**, the C15–C29 section of the molecule exists in multiple conformations as evident by the presence of multiple peaks for the methyl singlets of C14, and doublets of C30–C34 (see second set of singlets at 2.08, 1.95, 1.62 ppm, and doublets at 0.82 and 0.79 ppm within the ^1^H NMR spectrum of **1** in the Supporting Information). This was further supported by the presence of cross peak duplication in the ^1^H-^13^C HMBC spectrum, and exchange peaks in the ^1^H-^1^H NOESY spectrum suggesting that at least two conformations were present.

While the polyketide synthase (PKS) derived region (C15–C29 and C30–C34) remained uniform, we soon discovered that the alkoxy-2-propanone (C35-C37 in **1**, Figure 3a) was unstable and led to the formation of traces of **2** (Supporting Figure 2). This observation was further supported by the increase in the hydrolysis of **1** to **2** upon storage in CD_3_OD solution during NMR studies (data not shown). While methods were established to enable assignment of the aromatic resonances for rifamycin B (Supporting Table 3) and rifamycin S (Supporting Table 4), complete assignment of the aromatic resonances of **1** was not possible. The data reported in Supporting Tables 3 and 4 were collected for samples of rifamycin B or S at comparable concentrations as **1** in CD_3_OD (note the inclusion of ^13^C NMR spectra and peaks within the aryl region of the ^1^H-^13^C HMBC for these two compounds).

With the carbon scaffold identified, we turned our attention to evaluate the stereochemistry of **1** by interpretation of ^1^H-^1^H NOESY NMR data (Figure 3d,e, Supporting Table 2). Fortunately, a wealth of X-ray crystal structures of rifamycin analogues exists in the CDCC database. Using the crystal structure of rifampicin methanol solvate trihydrate (deposition CDC 859793), we systematically compared the observed NOEs (Supporting Figure 3) and coupling constants (Supporting Figure 4) with the configuration observed in the crystal structure data (Figure 3d,e) and found them consistent with a few exceptions. First, in solution, the olefinic protons at C28 and C29 interact with protons on both sides of the molecule, presumably because of free rotation around the C27–C28 and C29–O bonds. Similar rotation was observed between the diene from C16–C19. Nevertheless, the NOESY data not only confirmed the stereochemical assignment of the PKS portion of the molecule but also confirmed the full stereostructure assigned to **1**.

## Discussion

*S. aureus* is a leading cause of global mortality due to the increase in antibiotic resistant strains,^23^ and new antibiotics are urgently needed. Using our new 3-color THP-1 macrophage fluorescence imaging assay for intracellular *S. aureus* inhibition screening in iterative combination with nanomole-scaled techniques, we were able to identify a rifamycin analogue **1** that has not yet been described. The structure of **1** was shown to have a unique and readily activated alkyoxypropan-2-one motif. Samples of purified **1** were tested at a range of concentrations in our infection assay against *S. aureus* infected and non-infected differentiated THP-1 cells, and the results are shown in Figure 2b. The cell count of non-infected THP-1 cells suggested that although compound **1** was non-toxic at lower concentrations, toxicity was detected at 50 μM. Furthermore, cell count for infected cells indicates that **1** is most active at 1 and 10 μM. Combining activity and toxicity suggest that compound **1** has a narrow activity window of >1 and <50 μM. Given the clinical use of rifamycins to treat tuberculosis,^24^ we decided to evaluate the activity of **1** against *M. tuberculosis*. Using the microplate Alamar blue assay (MABA) and low oxygen recovery assay (LORA),^25^**1** demonstrated activity against *M. tuberculosis* with MIC values of 1.3 and 16.8 μM, respectively. Parallel MABA analyses indicated that this compares favorably to rifamycin O (0.093 μM LORA), rifamycin SV (0.044 μM LORA), and rifampicin (0.13 μM LORA or 0.024 μM MABA). While active, the reduced activity of **1** is not unexpected given its need for oxidative activation to **2** (Supporting Figure 2).

## Conclusions

With the first three-color intra-macrophage infection assay for *S. aureus* developed and demonstrated, the stage is set for larger high-throughput screens of small molecule libraries and for the identification of novel natural products from extract screening. With the ultimate need to develop new antibacterial drugs to tackle the global AMR crisis, we expect that this protocol will be adapted for general use as it allows the identification of compounds that can target intracellular bacteria. As described herein for *S. aureus* and by Brodin and Christophe^5^ for *M. tuberculosis*, the use of intracellular assays offers an important next step in identifying cell viable, and hence active, hits.

Marine actinobacteria are known for their prolific production of secondary metabolites,^26^ many of which have demonstrated clinical utility.^27^ Access to sequence data and new genomic tools has revolutionized our ability to analyze and manipulate large complex biosynthetic gene clusters (BGCs).^28^ This capability has advanced to the point where it is now possible to refactor old drugs for better treatments of infectious diseases.^29^ Here, we applied a new screening approach to an unexplored, marine rifamycin producer, *S. arenicola*,^20^ to gain access to a new class of rifamycin analogues.^30−32^ On-going sequencing efforts on strains of *Amycolatopsis* and *Salinispora* illustrate the complex divergence within the biosynthesis of rifamycin observed in strains of *Amycolatopsis* and *Salinispora* (Supporting Figure 5). While the PKS portion of this pathway retains a common overall gene architecture (Supporting Figure 5a), a high degree of divergence is apparent within the pre-PKS supply (Supporting Figure 5b) and post-PKS tailoring enzymes (Supporting Figure 5d). This divergence suggests that other, yet unexplored, rifamycin analogues, particularly those obtained through post-PKS modifications, could also exist.^20^ Successful capture of these modifications through comparative genomic analyses provides an excellent opportunity to blueprint a minimal rifamycin synthase with the goal of delivering new clinical agents. Here, the tremendous wealth of *Salinispora* genomes provides an excellent foundation to launch such efforts.^33^

The rifamycin group of antibiotics has demonstrated efficacy against bacterial and mycobacterial infections marked by its critical applications in the treatment of tuberculosis,^34^ leprosy,^35^ and Traveler’s diarrhea.^36^ Rifamycin polyketides are one of the few drug classes active against replicating and non-replicating bacteria, and new leads are critical to further clinical improvements. A recent survey of a series of strains of *S. arenicola* (Supporting Figure 6) indicated that extracts from 10 selected strains produced rifamycins, whose structure and functionality has yet to be explored. Here, we demonstrate how the combination of a new assay and miniaturized isolation methods can effectively direct identification of new natural products as starting points for the development of new drugs.

## Methods

### Preparation of THP-1 Macrophages

THP-1 cells (kind gift from Prof. David Hume) were maintained in RPMI-1640 medium (Thermo Fisher) supplemented with 10% heat inactivated fetal bovine serum (FBS, Life Technologies) and GlutaMax (Thermo Fisher) at 37 °C in a 5% CO_2_ environment. Preparation of THP-1 cells prior to infection, THP-1 monocytes were differentiated into macrophages by treatment with 50 nM phorbol 12-myristate 13-acetate (PMA, VWR) for 3 days at a density of 5 × 10^4^ cells per well in a 96-well tissue culture plate (Thermo Fisher, Nunclon Delta). The media was then replaced with fresh PMA-free media, and the cells were left to rest for 24 h before the infection protocol. Cell differentiation following PMA stimulation was monitored over time by microscopic observation of cell morphology and plastic adherence properties.

### Macrophage Infection with GFP-*S. aureus*

The *S. aureus* USA300 LAC strain that constitutively expresses GFP under a sarA-P1 promoter inserted markerless in the bacterial chromosome^14^ was used in this study. Bacterial overnight culture in tryptone soy broth (TSB, Oxoid) was sub-cultured in fresh TSB (1:100 dilution) at 37 °C, 200 rpm until reaching an OD_600_ value of 0.6. The bacteria solution was pelleted, washed, and suspended in macrophage culture media to a concentration of 5 × 10^6^ bacteria/mL. A 100 μL aliquot of the bacterial solution was added to the THP-1 macrophage culture, and the plate was centrifuged at 300 × *g* for 5 min to facilitate the macrophage–bacteria interaction. The infection was maintained for 1 h at 37 °C in a 5% CO_2_ atmosphere. Afterward, the media was replaced with media containing 100 μg/mL of gentamicin (Sigma, G1397) and left to incubate for 30 min to kill extracellular bacteria. The supernatant was then removed, and the cells were washed twice with fresh media prior to addition of the test fractions.

### Addition of Test Fractions

A total of 108 marine microbial test fractions containing ∼5 mg/mL of dry fraction in DMSO (Sigma–Aldrich) were screened. For the screen, each fraction was diluted in macrophage culture media at 200× and 1000× (corresponding to 0.025 and 0.005 mg/mL, respectively) and added to the thoroughly washed THP-1 macrophages at total volume of 200 μL/well, and the plate was left in the incubator overnight (16 h). Equivalent amount of DMSO was added to the cells in the DMSO control wells. Comparable methods were applied to test fractions and purified samples of **1**.

### Cell Fixation and Staining

THP-1 macrophages were then fixed with 4% formaldehyde (formaldehyde 16% MeOH free, ThermoFisher Scientific) in phosphate buffered saline (PBS) for 20 min at 23 °C and subsequently washed thoroughly in PBS. Next, the cell membranes and the nuclei of the THP-1 macrophages were stained with Alexa Fluor 555 conjugated wheat germ agglutinin (ThermoFisher Scientific) and Hoechst 33342 (ThermoFisher Scientific) in PBS according to the supplier’s protocols. The cells were then washed twice thoroughly in PBS, a final volume of 100 μL of PBS was added to each well, and the plate was stored at 4 °C until imaging.

### Imaging Assays

Imaging was performed using the Opera HCS (PerkinElmer) instrument. Two independent exposures with two fluorescence channels each were used to avoid bleed through. Exposure 1 parameters: laser excitation 488 nm at 2.5 mW, emission filter 520/35 nm. Exposure 2 parameters: laser excitation 561 nm at 2 mW and emission filter 585/40 nm and UV excitation at 365 nm and emission filter 450/50 nm, respectively. The exposure time for the three laser channels was 240 ms, and the UV exposure time was 20 ms. All imaging was performed using a 20× Air LUCPLFLN objective (Olympus) with a NA of 0.45, and 20 images per well were captured.

### Image Data Analyses

Image analysis was completed using the image analysis software Acapella (PerkinElmer). Two versions of the script were developed, one that is compatible with the Opera instrument to run while imaging (version 2.7) and one supporting instruments with Harmony and Windows 10 (version 5.2+). The script was calibrated using different treatment conditions: positive (50 nM rifampicin) and negative control (no rifampicin) treated wells, of *S. aureus* infected THP-1 macrophages and non-infected cells (Figure 1c). The workflow is described in the algorithm flowchart, and example images are shown as well (Supporting Figure 7). Briefly, nuclei were detected using the algorithm H in the Hoechst channel, and border objects were removed to ensure only whole nuclei were analyzed. Area, roundness, and intensity were calculated to filter objects downstream of the analysis. The cytoplasm was detected using algorithm B in the macrophage marker (cytoplasm channel). Those areas were kept as regions of interest to define individual cell areas. For those regions, properties such as area and intensity and their standard deviations were calculated. A “find spots” algorithm was utilized for the infectious bacteria (*S. aureus* GFP channel) foci analysis. Staining intensity and morphological properties were calculated in all channels, and infectious ratio (normal cell fragmentation) was calculated for the cells identified (Supporting Table 6). Potential artifacts in the analysis were removed using cell-like property filters. The remaining cell-like objects were transferred into a CSV output file. Final data analysis and graph plotting were performed in Excel and Prism (GraphPad).

### Development of Assay Parameters

Initially, the assay was tested with a range of mode of multiplicity to find the optimum. At MOI of 5, and below, the outcome of the assay was random. For example, when infecting cells in 60 wells at MOI 5, a significant number of wells show a large number of live macrophages remaining after the overnight incubation (without any addition of a test fraction or purified **1**) whereas in the remaining wells, there were no live macrophages. Hence, the number of remaining live macrophages followed a stochastic distribution making the assay not useful. Therefore, we chose MOI of 10 to obtain a stable assay. To further characterize the assay, *S. aureus* infected and non-infected differentiated THP-1 cells were kept overnight in complete RPMI-1640 medium with and without 50 nM rifampicin (Figure 1d). After washing, fixating, staining, imaging, and image analysis to identify the number of remaining THP-1 cells, dead cells were washed away during the fixing and staining procedures. These results are shown in Figure 1. For non-infected THP-1 cells in the presence or absence of 50 nM rifampicin, there are a significant number of cells left after the incubation (Figure 1d). For infected THP-1 cells in the absence of rifampicin (50 nM), cell death was predominant as noted by the presence of only a few cells after washing and fixation. However, in the presence of 50 nM rifampicin, there were a significant number of infected cells left, comparable to the numbers for the non-infected cells. This shows that our assay was sensitive with a large separation between our positive control (50 nM rifampicin) and negative control (cell media). However, it was difficult to use the negative control, due to the number of remaining cells being very close to zero, to define the threshold for hit identification. Hence, we chose the lower three-sigma threshold (three standard deviation) or a Z-score > −3 of the average cell number of the infected THP-1 cells in the presence of 50 nM rifampicin (red line in Figure 1d) as our hit threshold, the minimum cell number a test fraction needed to be classified as a hit.

### Assay-Guided Purification of **1**

A 100 μL sample of the test fraction in DMSO was dried by air flow, redissolved in MeOH (50 μL), applied to a pTLC plate (5 cm × 20 cm), and eluted with a 1:9 mixture of MeOH:EtOAc. Eight bands (Y014F9-1 to Y014F9-8) were obtained and submitted to NMR analyses. Each of these fractions was rescreened to identify Y014F9-6 as the active fraction. ^1^H NMR spectra were collected on a sample of each fraction. The remaining Y014F9-6 fraction was then redissolved in MeOH (50 μL) and applied to a pTLC plate (5 cm × 20 cm) and eluted with a 1:9 mixture of MeOH:EtOAc to deliver three fractions Y014F9-6A, Y014F9-6B, and Y014F9-6C. The second repetition assay identified Y014F9-6B as the most active fraction.^1^H NMR spectra were collected on a sample of each fraction. Comparable methods were used to screen the activity of purified **1**.

### Reculturing and Fractionation of *S. arenicola* CNY-014

*S. arenicola* CNY-014 strain was cultured in 5 L scale for 7 days with shaking at 180 RPM. After this period, the cultures were extracted with EtOAc (2 × 5 L) and the extract was dried with Na_2_SO_4_ and concentrated by rotary evaporation to yield 400 mg of organic extract. The ethyl acetate extract (400 mg) was subjected to silica chromatography, using a step gradient solvent system of *n*-hexane, EtOAc, and MeOH (1:0:0, 4:1:0, 3:2:0, 1:1:0, 1:4:0, 0:1:0, 0:9:1, 0:1:1, and 0:0:1) to yield 10 fractions as provided in Supporting Table 1.

### Reisolation from *S. arenicola* CNY-014

Pure **1** was obtained from the CNY014-S10 fraction by pTLC purification. The S10 fraction was dissolved in 100 μL of MeOH, applied to a pTLC plate (5 cm × 20 cm), and eluted with a 1:9 mixture of MeOH:EtOAc. Six bands (CNY014-S10A to CNY014-S10F) were obtained. Compound **1** was found in fraction CNY014-S10E. Repetition of the pTLC purification eluting with a 1:10 mixture of MeOH:acetone provided pure **1** (580 μg). This material was used for NMR analyses and structure elucidation studies. Samples of rifamycin B (Sigma–Aldrich, SIAL-R0900000) and rifamycin S (TCI America, R02001G) were obtained commercially to assist in the structure elucidation effort.

### Structure Elucidation of **1**

NMR data were acquired with a Bruker Avance III 600 MHz spectrometer equipped with a 1.7 mm cryoprobe. Chemical shifts were referenced using the corresponding solvent signals (δ_H_ 7.26 and δ_C_ 77.0 for CDCl_3_ and δ_H_ 3.31 and δ_C_ 49.0 for CD_3_OD). The NMR spectra were processed using Mnova 11.0 (Mestrelab Research) or TopSpin 3.6 (Bruker Biospin) software.

## Supporting Information Available

Tabulation of the fractionation methods; tabulated NMR data for rifamycin analogue **1**, rifamycin B, and rifamycin S; tabulated NMR data to compare rifamycin analogue **1** to rifamycin B and rifamycin S; parameters used for image analysis using Acapella; NMR peak shift analyses; schematic representation of the degradation of **1**; NOE and coupling constant depiction for **1**; comparison of the rifamycin BGCs from *A. mediterranei* and *S. arenicola*; comparative evaluation of the levels of **1** in additional strains of *S. arenicola*; and additional description of the Acapella image analyses (PDF)

## References

[ref1] Tommasi R.; Brown D. G.; Walkup G. K.; Manchester J. I.; Miller A. A. ESKAPEing the labyrinth of antibacterial discovery. Nat. Rev. Drug Discov. 2015, 14, 529–542. 10.1038/nrd4572. 26139286

[ref2] Silver L. L. Challenges of antibacterial discovery. Clin. Microbiol. Rev. 2011, 24, 71–109. 10.1128/CMR.00030-10. 21233508 PMC3021209

[ref3] Payne D. J.; Gwynn M. N.; Holmes D. J.; Pompliano D. L. Drugs for bad bugs: confronting the challenges of antibacterial discovery. Nat. Rev. Drug Discov. 2007, 6, 29–40. 10.1038/nrd2201. 17159923

[ref4] Lewis K. Platforms for antibiotic discovery. Nat. Rev. Drug Discov. 2013, 12, 371–387. 10.1038/nrd3975. 23629505

[ref5] Brodin P.; Christophe T. High-content screening in infectious diseases. Curr. Opin. Chem. Biol. 2011, 15, 534–539. 10.1016/j.cbpa.2011.05.023. 21684803

[ref6] Christophe T.; Jackson M.; Jeon H. K.; Fenistein D.; Contreras-Dominguez M.; Kim J.; Genovesio A.; Carralot J. P.; Ewann F.; Kim E. H.; Lee S. Y.; Kang S.; Seo M. J.; Park E. J.; Skovierová H.; Pham H.; Riccardi G.; Nam J. Y.; Marsollier L.; Kempf M.; Joly-Guillou M. L.; Oh T.; Shin W. K.; No Z.; Nehrbass U.; Brosch R.; Cole S. T.; Brodin P. High content screening identifies decaprenyl-phosphoribose 2′ epimerase as a target for intracellular antimycobacterial inhibitors. PLoS Pathog. 2009, 5, e1000645 10.1371/journal.ppat.1000645. 19876393 PMC2763345

[ref7] Pethe K.; Bifani P.; Jang J.; Kang S.; Park S.; Ahn S.; Jiricek J.; Jung J.; Jeon H. K.; Cechetto J.; Christophe T.; Lee H.; Kempf M.; Jackson M.; Lenaerts A. J.; Pham H.; Jones V.; Seo M. J.; Kim Y. M.; Seo M.; Seo J. J.; Park D.; Ko Y.; Choi I.; Kim R.; Kim S. Y.; Lim S.; Yim S. A.; Nam J.; Kang H.; Kwon H.; Oh C. T.; Cho Y.; Jang Y.; Kim J.; Chua A.; Tan B. H.; Nanjundappa M. B.; Rao S. P.; Barnes W. S.; Wintjens R.; Walker J. R.; Alonso S.; Lee S.; Kim J.; Oh S.; Oh T.; Nehrbass U.; Han S. J.; No Z.; Lee J.; Brodin P.; Cho S. N.; Nam K.; Kim J. Discovery of Q203, a potent clinical candidate for the treatment of tuberculosis. Nat. Med. 2013, 19, 1157–1160. 10.1038/nm.3262. 23913123

[ref8] Ellis M. J.; Tsai C. N.; Johnson J. W.; French S.; Elhenawy W.; Porwollik S.; Andrews-Polymenis H.; McClelland M.; Magolan J.; Coombes B. K.; Brown E. D. A macrophage-based screen identifies antibacterial compounds selective for intracellular *Salmonella typhimurium*. Nat. Commun. 2019, 10, 197 10.1038/s41467-018-08190-x. 30643129 PMC6331611

[ref9] Guiguemde W. A.; Shelat A. A.; Bouck D.; Duffy S.; Crowther G. J.; Davis P. H.; Smithson D. C.; Connelly M.; Clark J.; Zhu F.; Jiménez-Díaz M. B.; Martinez M. S.; Wilson E. B.; Tripathi A. K.; Gut J.; Sharlow E. R.; Bathurst I.; El Mazouni F.; Fowble J. W.; Forquer I.; McGinley P. L.; Castro S.; Angulo-Barturen I.; Ferrer S.; Rosenthal P. J.; Derisi J. L.; Sullivan D. J.; Lazo J. S.; Roos D. S.; Riscoe M. K.; Phillips M. A.; Rathod P. K.; Van Voorhis W. C.; Avery V. M.; Guy R. K. Chemical genetics of *Plasmodium falciparum*. Nature 2010, 465, 311–315. 10.1038/nature09099. 20485428 PMC2874979

[ref10] Pidwell G. R.; Gibson J. F.; Cole J.; Renshaw S. A.; Foster S. J. The role of macrophages in *Staphylococcus aureus* infection. Front Immunol. 2021, 11, 620339 10.3389/fimmu.2020.620339. 33542723 PMC7850989

[ref11] Peyrusson F.; Varet H.; Nguyen T. K.; Legendre R.; Sismeiro O.; Coppée J. Y.; Wolz C.; Tenson T.; Van Bambeke F. Intracellular *Staphylococcus aureus* persisters upon antibiotic exposure. Nat. Commun. 2020, 11, 2200 10.1038/s41467-020-15966-7. 32366839 PMC7198484

[ref12] Meylan S.; Andrews I. W.; Collins J. J. Targeting antibiotic tolerance, pathogen by pathogen. Cell 2018, 172, 1228–1238. 10.1016/j.cell.2018.01.037. 29522744

[ref13] de Jong N. W.; van der Horst T.; van Strijp J. A.; Nijland R. Fluorescent reporters for markerless genomic integration in *Staphylococcus aureus*. Sci. Rep. 2017, 7, 43889 10.1038/srep43889. 28266573 PMC5339689

[ref14] Dubois J.; Dubois M. J. Levonadifloxacin (WCK 771) exerts potent intracellular activity against *Staphylococcus aureus* in THP-1 monocytes at clinically relevant concentrations. J. Med. Microbiol. 2019, 68, 1716–1722. 10.1099/jmm.0.001102. 31689174

[ref15] Garcia R. C.; Banfi E.; Pittis M. G. Infection of macrophage-like THP-1 cells with Mycobacterium avium results in a decrease in their ability to phosphorylate nucleolin. Infect. Immun. 2000, 68, 3121–3128. 10.1128/IAI.68.6.3121-3128.2000. 10816453 PMC97542

[ref16] Thomas F.; Pittman K.; Dabney L.; Bogey W.; Clark G.; DeMasi R. Studies of radioimmunoreactive urinary insulin (RUI) in the human: a new test for diagnosis of pancreas rejection. Transplant. Proc. 1989, 21, 2797–2798. 2650366

[ref17] Shiratori H.; Feinweber C.; Luckhardt S.; Linke B.; Resch E.; Geisslinger G.; Weigert A.; Parnham M. J. THP-1 and human peripheral blood mononuclear cell-derived macrophages differ in their capacity to polarize in vitro. Mol. Immunol. 2017, 88, 58–68. 10.1016/j.molimm.2017.05.027. 28600970

[ref18] Han H. W.; Seo H. H.; Jo H. Y.; Han H. J.; Falcão V. C. A.; Delorme V.; Heo J.; Shum D.; Choi J. H.; Lee J. M.; Lee S. H.; Heo H. R.; Hong S. H.; Park M. H.; Thimmulappa R. K.; Kim J. H. Drug discovery platform targeting *M. tuberculosis* with human embryonic stem cell-derived macrophages. Stem Cell Rep. 2019, 13, 980–991. 10.1016/j.stemcr.2019.10.002. PMC6915848 31680058

[ref19] Hughes C. C.; Fenical W. Antibacterials from the sea. Chemistry 2010, 16, 12512–12525. 10.1002/chem.201001279. 20845412 PMC3071975

[ref20] Kim H.; Kim S.; Kim M.; Lee C.; Yang I.; Nam S. J. Bioactive natural products from the genus *Salinispora*: a review. Arch. Pharmacal Res. 2020, 43, 1230–1258. 10.1007/s12272-020-01288-1. 33237436

[ref21] Rinehart K. L. Jr.; Shield L. S. Chemistry of the ansamycin antibiotics. Fortschr. Chem. Org. Naturst. 1976, 33, 231–307. 10.1007/978-3-7091-3262-3_3. 11155

[ref22] Riva S.; Silvestri L. G. Rifamycins: a general view. Annu. Rev. Microbiol. 1972, 26, 199–224. 10.1146/annurev.mi.26.100172.001215. 4562808

[ref23] Global burden of bacterial antimicrobial resistance in 2019: a systematic analysis. Lancet 2022, 399, 629–655. 10.1016/S0140-6736(21)02724-0. 35065702 PMC8841637

[ref24] Alfarisi O.; Alghamdi W. A.; Al-Shaer M. H.; Dooley K. E.; Peloquin C. A. Rifampin *vs*. rifapentine: what is the preferred rifamycin for tuberculosis?. Expert Rev. Clin. Pharmacol. 2017, 10, 1027–1036. 10.1080/17512433.2017.1366311. 28803492

[ref25] Cho S.; Lee H. S.; Franzblau S. Microplate Alamar Blue Assay (MABA) and Low Oxygen Recovery Assay (LORA) for *Mycobacterium tuberculosis*. Methods Mol. Biol. 2015, 1285, 281–292. 10.1007/978-1-4939-2450-9_17. 25779323

[ref26] Jensen P. R.; Williams P. G.; Oh D. C.; Zeigler L.; Fenical W. Species-specific secondary metabolite production in marine actinomycetes of the genus *Salinispora*. Appl. Environ. Microbiol. 2007, 73, 1146–1152. 10.1128/AEM.01891-06. 17158611 PMC1828645

[ref27] Chu L.; Huang J.; Muhammad M.; Deng Z.; Gao J. Genome mining as a biotechnological tool for the discovery of novel marine natural products. Crit. Rev. Biotechnol. 2020, 40, 571–589. 10.1080/07388551.2020.1751056. 32308042

[ref28] Lammens E. M.; Nikel P. I.; Lavigne R. Exploring the synthetic biology potential of bacteriophages for engineering non-model bacteria. Nat. Commun. 2020, 11, 5294 10.1038/s41467-020-19124-x. 33082347 PMC7576135

[ref29] Li L.; Maclntyre L. W.; Brady S. F. Refactoring biosynthetic gene clusters for heterologous production of microbial natural products. Curr. Opin. Biotechnol. 2021, 69, 145–152. 10.1016/j.copbio.2020.12.011. 33476936 PMC8238852

[ref30] Kim T. K.; Hewavitharana A. L.; Sjaw P. N.; Fuerst J. A. Discovery of a New Source of Rifamycin Antibiotics in Marine Sponge Actinobacteria by Phylogenetic Prediction. Appl. Environ. Microbiol. 2006, 72, 2118–2125. 10.1128/AEM.72.3.2118-2125.2006. 16517661 PMC1393243

[ref31] Peek J.; Lilic M.; Montiel D.; Milshteyn A.; Woodworth I.; Biggins J. B.; Ternei M. A.; Calle P. Y.; Danziger M.; Warrier T.; Saito K.; Braffman N.; Fay A.; Glickman M. S.; Darst S. A.; Campbell E. A.; Brady S. F. Rifamycin congeners kanglemycins are active against rifampicin-resistant bacteria via a distinct mechanism. Nat. Commun. 2018, 9, 4147 10.1038/s41467-018-06587-2. 30297823 PMC6175910

[ref32] Zhou Q.; Luo G. C.; Zhang H.; Tang G. L. Discovery of 16-Demethylrifamycins by Removing the Predominant Polyketide Biosynthesis Pathway in *Micromonospora* sp. Strain TP-A0468. Appl. Environ. Microbiol. 2019, 85, e02597-18 10.1128/AEM.02597-18. 30530711 PMC6365818

[ref33] Jensen P. R.; Moore B. S.; Fenical W. The marine actinomycete genus *Salinispora*: a model organism for secondary metabolite discovery. Nat. Prod. Rep. 2015, 32, 738–751. 10.1039/C4NP00167B. 25730728 PMC4414829

[ref34] Tetali S. R.; Kunapaeddi E.; Mailavaram R. P.; Singh V.; Borah P.; Deb P. K.; Venugopala K. N.; Hourani W.; Tekade R. K. Current advances in the clinical development of anti-tubercular agents. Tuberculosis 2020, 125, 101989 10.1016/j.tube.2020.101989. 32957054

[ref35] Richardus J. H.; Tiwari A.; Barth-Jaeggi T.; Arif M. A.; Banstola N. L.; Baskota R.; Blaney D.; Blok D. J.; Bonenberger M.; Budiawan T.; Cavaliero A.; Gani Z.; Greter H.; Ignotti E.; Kamara D. V.; Kasang C.; Manglani P. R.; Mieras L.; Njako B. F.; Pakasi T.; Pandey B. D.; Saunderson P.; Singh R.; Smith W. C. S.; Stäheli R.; Suriyarachchi N. D.; Tin Maung A.; Shwe T.; van Berkel J.; van Brakel W. H.; Vander Plaetse B.; Virmond M.; Wijesinghe M. S. D.; Aerts A.; Steinmann P. Leprosy post-exposure prophylaxis with single-dose rifampicin (LPEP): an international feasibility programme. Lancet Glob. Health 2021, 9, e81–e90. 10.1016/S2214-109X(20)30396-X. 33129378

[ref36] Hoy S. M. Rifamycin SV MMX: A review in the treatment of Traveller’s Diarrhoea. Clin. Drug Investig. 2019, 39, 691–697. 10.1007/s40261-019-00808-2. 31172447

