# Supplementary material for: Nanoscaled Discovery of a Shunt Rifamycin from Salinispora arenicola Using a Three-Color GFP-Tagged Staphylococcus aureus Macrophage Infection Assay
Source: ACS Infect Dis. 2023 Jul 11;9(8):1499–507. doi: 10.1021/acsinfecdis.3c00049 (PMC10425972; doi:10.1021/acsinfecdis.3c00049)

Supporting Information

Nanoscaled discovery of a shunt rifamycin from *Salinispora arenicola* using a three-colour GFP-tagged *Staphylococcus aureus* macrophage infection assay

Nhan T. Pham,[1] Joana Alves,[2] Fiona A. Sargison,[2] Reiko Cullum,[3] Jan Wildenhain,[4] William Fenical,[3,7] Mark S. Butler,[5] David A. Mead,[6] Brendan M. Duggan,[7] J. Ross Fitzgerald,*[,2] James J. La Clair,*[,5,8] & Manfred Auer[*,1,5]

[1] School of Biological Sciences, The University of Edinburgh, The King's Buildings, Edinburgh EH9 3BF, United Kingdom.

[2] The Roslin Institute, The University of Edinburgh, Easter Bush Campus, Midlothian EH25 9RG, United Kingdom

[3] Center for Marine Biotechnology and Biomedicine, Scripps Institution of Oceanography, University of California at San Diego, La Jolla, California, 92093-0204, United States.

[4] Exscientia Oxford Science Park, The Schrödinger Building, Oxford Science Park, The Schrödinger Building, Oxford OX4 4GE, United Kingdom

[5] Xenobe Research Institute, P. O. Box 3052, San Diego, California, 92163, United States

[6] Terra Bioforge Inc, 3220 Deming Way Suite 100, Middleton, WI 53562, United States

[7] Skaggs School of Pharmacy and Pharmaceutical Sciences, University of California, San Diego, 9500 Gilman Drive, La Jolla, California, 92093, United States

[8] Department of Chemistry and Biochemistry, University of California at San Diego, La Jolla, California, 92093-0358, United States

[*] Corresponding authors: ross.fitzgerald@ed.ac.uk (R. F), i@xenobe.org (J. J. L), or manfred.auer@ed.ac.uk (M. A.)

Table of Contents **Page**

Table of Contents	Page
Supporting Table 1	S3
Supporting Table 2	S4
Supporting Table 3	S5
Supporting Table 4	S6
Supporting Table 5	S7
Supporting Table 6	S8
Supporting Figure 1	S9
Supporting Figure 2	S10
Supporting Figure 3	S11
Supporting Figure 4	S12
Supporting Figure 5	S13
Supporting Figure 6	S14
Supporting Figure 7	S15
Additional Spectral Data	S16-S31

Supporting Table 1. Fractionation method and materials obtained.

Initial fractionation

fraction	wt (mg)	hexanes	EtOAc	MeOH
Y014F9-1	0.5	100%	-	-
Y014F9-2	0.8	66	33	-
Y014F9-3	1.2	50	50	-
Y014F9-4	1.1	20	80	-
Y014F9-5	1.4	-	100	-
Y014F9-6	1.2	-	90	10
Y014F9-7	1.9	-	50	50
Y014F9-8	0.3	-	-	100

One half of the Y014F9-6 was used for rescreening and the second half was further purified by preparative TLC to afford sub-fractions Y014F9-6A, Y014F9-6B, and Y014F9-6C. Due to their small-scale reliable masses were not determined at this stage.

Fractionation used in reculturing

	wt (mg)	hexanes	EtOAc	MeOH
S01	3	100%	-	-
S02	6	80	20	-
S03	26	60	40	-
S04	27	50	50	-
S05	33	20	80	-
S06	27	-	100	-
S07	22	-	90	10
S08	168	-	50	50
S09	25	-	-	100
S10	8	-	-	100

Supporting Table 2. NMR data for rifamycin analogue **1** in CD_3OD

	δ_H, mult (*J* in Hz)	δ_C[1,2]	$^1H,^1H$ gCOSY	$^1H,^1H$ NOESY[3]	$^1H,^{13}C$ HMBC
1		ND[4]			
2		ND[4]			
3		ND[4]			
4		ND[4]			
5		ND[4]			
6		172.0			
7		108.6			
8		173.5[6]			
9		ND[4]			
10		ND[4]			
11		198.4			
12		109.1			
13	1.67, s	21.7	–	–	11,12
14	2.10, s	6.8	–	–	6,7,8
15		171.7			
16		124.8			
17	6.21, d (11.5)	133.6	18,30	19,30	15w,19,30
18	7.11, dd (16.0, 10.7)	128.5	17,19	21,31	17,20w
19	6.07, dd (16.2, 7.4)	139.6	18,20	17,31w	17,20w
20	2.34, dt (9.9, 7.0)	38.6	19w,21,31	32	19w
21	4.05, d (11.2)	75.6	20,22	18,24,31,33	32
22	1.91, m	34.2	21w,23w,32	31,33	–
23	3.46, dd (10.3, 2.5)	78.4	22,24	25w,32,33	21w
24	1.56, m	39.8	23,33	21,34	–
25	3.71, d (10.7)	72.2	26	23,27w,33w,34	23w,26w,33
26	1.33, m	42.2	25,34	33	–
27	4.42, d (7.0)	68.3	26,28,29w	25,29,34w	26,29,34
28	5.31, dd (12.7, 7.0)	124.9	27,29	26,34	29
29	6.24, dd (12.7, 1.4)	141.9	27w,28	26,27,34	27w, 28
30	2.01, s	20.5	–	–	15,17
31	0.98, d (6.7)	18.3	20	21,22	19,20,21
32	1.05, d (6.9)	10.8	22	20,23	21,22,23
33	0.74, d (6.9)	9.1	24	21,22,23,26	23,24,25
34	-0.05, d (6.7)	9.9	26	24,25,28w	25,26,27
35	3.04, m	56.5		37	36,37
36		208.8			
37	2.29, s	32.9		35	36
NH	8.54, s[5]				

[1,2] Assignments obtained [1] from $^1H,^{13}C$ HSQC or [2] from $^1H,^{13}C$ HMBC spectra.
3 NOEs from protons on neighbouring carbons are not tabulated.
[4] Not determined. Sample sizes were too small to collect directly detected ^{13}C NMR spectra, nor were we able to obtain sufficient sensitivity to assign these carbons from $^1H,^{13}C$ HMBC spectra. This was further complicated by the fact that the ketone at C36 could exist as a hemiketal with the OH group at C3 resulting in 3 tautomers (1 ketone and two ketal stereoisomers) from this position. This tautomerization along with the low sample size prevented assignment at C1-C5.
[5] This assignment was tentative and could arise from an impurity such as formate
[6] This chemical shift likely arose from the degradation of **1** to **2** as shown in Supporting Fig. S2.

Supporting Table 3. NMR data for rifamycin B in CD_3OD

	δ_H, mult (*J* in Hz)	δ_C	$^1H,^1H$ gCOSY	$^1H,^{13}C$ HMBC
1		147.9[2]		
2		123.4[2]		
3	7.37, bs	109.1		–
4		147.9[1]		
5		103.5[2]		
6		159.7[2]		
7		107.6[2]		
8		119.0[2]		
9		107.6[2]		
10		115.3[2]		
11		175.4[1]		
12		110.3[1]		
13	1.76, s	22.2		12
14	2.19, s	7.6		7,9,11
15		172.7[1]		
16		132.1[1]		
17	6.31, d (10.5)	134.2	18,30w	15,18w,19w,30
18	6.43, dd (15.6, 10.9)	125.3	17,19	17w,20
19	6.01, dd (15.5, 6.0)	141.7	18,20	17,20w,21
20	2.34, m	39.6	19w,21,31	18w,19w,21w,31w
21	3.86, d (8.8)	73.6	20,22w	19w,20w,32
22	1.78, m	34.4	32	23w,32
23	3.09, dd (10.2, 2.2)	78.1	22w,24	21,22w,24
24	1.45, dq (13.9, 7.2)	39.1	23,33	23.33
25	5.12, d (10.7)	74.9	26	23w,24w,26,33,34,35
26	1.20, m	41.0	25,34	25w
27	3.43, d (7.0)	78.5	28	25,26,28,29,34,37
28	5.08, dd (12.7, 7.0)	119.6	27,29	27w,29
29	6.20, dd (12.7, 1.0)	144.0	28	12,27,28
30	2.09, s	20.4	17w	15,16,17,18w,19w
31	0.92, d (7.0)	17.9	20	20,21
32	0.99, d (7.0)	11.4	22	21,22,23
33	0.60, d (6.9)	9.4	24	23,24,25
34	-0.34, d (6.9)	9.4	26	25.26.27
35		172.6[1]		
36	2.02, s	20.8		35
37	3.03, s	57.1	–	27
38	4.74, s	67.7		4,39
39		171.9[1]		
NH	7.92, d (0.6)			

[1] Assignments obtained from $^1H,^{13}C$ HMBC spectrum
[2] Assignments based on chemical shift predictions and literature precedent

Supporting Table 4. NMR data for rifamycin S in CD_3OD[3]

	δ_H, mult (*J* in Hz)	δ_C	$^1H, ^1H$	$^1H, ^{13}C$ HMBC
1		184.0[1,2]		
2		141.2[2]		
3	7.63, s	118.3	–	2,4,5,10
4		185.9[1.2]		
5		112.2[1,2]		
6		168.0[1,2]		
7		117.0[1,2]		
8		173.9[1.2]		
9		112.0[1,2]		
10		131.9[1,2]		
11		193.9[1]		
12		109.5[1]		
13	1.70, s	22.2	–	11,12,29w
14	2.31,s	7.7	–	1w,5,6,7,8,9,10
15		172.0[1]		
16		132.3[1]		
17	6.30, d (10.5)	134.3	18,30	15,19,30
18	6.26, m	125.8	17,19	16,17,20
19	5.91, dd (14.3, 6.5)	142.2	18,20	15w,17,20,21,30w,31
20	2.33, m	40.1	19w,21,31	18,19,21,31
21	3.69, dd (9.8, 1.9)	74.5	20,22w	19,20,21w,22w,23,32
22	1.78, m	34.0	21w,32	20w,23,32
23	3.10, d (2.1)	77.9	22w,24	21,22,24,32
24	1.51, m	38.7	23,33	22w,23,33
25	4.93, dd (10.5, 1.7)	73.9	24w,26	23,24,26,27,33,34w,35
26	1.83, dtd (10.4, 7.1,	38.8	25,27w,34	25,27w,28w,34
27	3.38, dd (7.9, 3.1)	82.3	26w,28	25,26,28,29,34,37
28	5.92, dd (12.6, 7.9)	118.1	27,29	12w,26w,27w,29
29	6.20, dd (12.6, 0.8)	145.6	28	12,27,28
30	2.04, s	20.2	17	15,16,17,18w,19w,31w
31	0.86, d (6.9)	17.5	20	20,21
32	0.99, d (7.0)	11.4	22	21,22,23
33	0.69, d (6.9)	9.3	24	23.24.25
34	0.09, d (7.1)	12.0	26	25,26,27
35		172.9[1]		
36	1.95, s	21.1	–	25w,35
37	3.08, s	56.9	–	27

[1] Assignments obtained from $^1H,^{13}C$ HMBC spectrum
[2] Assignments based on chemical shift predictions and literature precedent
[3] Contains impurity with 1.15, d (6.1), 25.3 and 3.93, h (6.2). 64.7

Supporting Table 5. Aligned ^{1}H and ^{13}C NMR data for **1**, rifamycin B, and rifamycin S in CD_3OD

	analogue **1**			rifamycin B		rifamycin S	
	δ_H, mult (*J* in Hz)	δ_C[1,2]		δ_H, mult (*J* in Hz)	δ_C[1,2]	δ_H, mult (*J* in Hz)	δ_C[1,2]
1		ND[3]	1		147.9		184.0
2		ND[3]	2		123.4		141.2
3		ND[3]	3	7.37, bs	109.1	7.63, s	118.3
4		ND[3]	4		147.9		185.9
5		ND[3]	5		103.5		112.2
6		172.0	6		159.7		168.0
7		108.6	7		107.6		117.0
8		173.5[4]	8		119.0		173.9
9		ND[3]	9		107.6		112.0
10		ND[3]	10		115.3		131.9
11		198.4	11		175.4		193.9
12		109.1	12		110.3		109.5
13	1.67, s	21.7	13	1.76, s	22.2	1.70, s	22.2
14	2.10, s	6.8	14	2.19, s	7.6	2.31,s	7.7
15		171.7	15		172.7		172.0
16		124.8	16		132.1		132.3
17	6.21, d (11.5)	133.6	17	6.31, d (10.5)	134.2	6.30, d (10.5)	134.3
18	7.11, dd (16.0, 10.7)	128.5	18	6.43, dd (15.6,	125.3	6.26, m	125.8
19	6.07, dd (16.2, 7.4)	139.6	19	6.01, dd (15.5, 6.0)	141.7	5.91, dd (14.3, 6.5)	142.2
20	2.34, dt (9.9, 7.0)	38.6	20	2.34, m	39.6	2.33, m	40.1
21	4.05, d (11.2)	75.6	21	3.86, d (8.8)	73.6	3.69, dd (9.8, 1.9)	74.5
22	1.91, m	34.2	22	1.78, m	34.4	1.78, m	34.0
23	3.46, dd (10.3, 2.5)	78.4	23	3.09, dd (10.2, 2.2)	78.1	3.10, d (2.1)	77.9
24	1.56, m	39.8	24	1.45, dq (13.9, 7.2)	39.1	1.51, m	38.7
25	3.71, d (10.7)	72.2	25	5.12, d (10.7)	74.9	4.93, dd (10.5, 1.7)	73.9
26	1.33, m	42.2	26	1.20, m	41.0	1.83, dtd (10.4,	38.8
27	4.42, d (7.0)	68.3	27	3.43, d (7.0)	78.5	3.38, dd (7.9, 3.1)	82.3
28	5.31, dd (12.7, 7.0)	124.9	28	5.08, dd (12.7, 7.0)	119.6	5.92, dd (12.6, 7.9)	118.1
29	6.24, dd (12.7, 1.4)	141.9	29	6.20, dd (12.7, 1.0)	144.0	6.20, dd (12.6, 0.8)	145.6
30	2.01, s	20.5	30	2.09, s	20.4	2.04, s	20.2
31	0.98, d (6.7)	18.3	31	0.92, d (7.0)	17.9	0.86, d (6.9)	17.5
32	1.05, d (6.9)	10.8	32	0.99, d (7.0)	11.4	0.99, d (7.0)	11.4
33	0.74, d (6.9)	9.1	33	0.60, d (6.9)	9.4	0.69, d (6.9)	9.3
34	-0.05, d (6.7)	9.9	34	-0.34, d (6.9)	9.4	0.09, d (7.1)	12.0
35	3.04, m	56.5	35		172.6		172.9
36		208.8	36	2.02, s	20.8	1.95, s	21.1
37	2.29, s	32.9	37	3.03, s	57.1	3.08, s	56.9
NH	8.54, s		38	4.74, s	67.7		
			39		171.9		
			NH	7.92, d (0.6)			

[1,2] Assignments [1]from ^{1}H,^{13}C HSQC or [2] from ^{1}H,^{13}C HMBC spectra as given in Supporting Tables 2-4.
[3] ND denotes assignments that were not detected.
[4] This chemical shift likely arose from the degradation of **1** to **2** as shown in Supporting Fig. S2.

Supporting Table 6. Parameters used for image analysis using Acapella

**General Parameters**	**Value**
Channel number for nuclear channel	4
Channel number for cytoplasm channel	3
Channel number for spot channel	1
Spot detection area	WholeCell
Remove Border Objects	Yes
Minimum roundness of cells (1 = perfect circle)	0.8
**Nuclei Detection**	
Nuclei Detection Algorithm	H
Threshold Adjustment	1
Minimum Nuclei Distance	10
Nuclear Splitting Adjustment	8
Individual Threshold Adjustment	0.45
Minimum Nuclear Area	100
Minimum Nuclear Contrast	0.3
**Nucleus Region**	
NucleusOuterBorderShift	0
NucleusInnerBorderShift	unlimited
**Cytoplasm Detection**	
Cytoplasm Threshold Adjustment	0.7
Cytoplasm Individual Threshold Adjustment	0.5
**Cytoplasm Region**	
CytoplasmOuterBorderShift	0
CytoplasmInnerBorderShift	0
NucleusBorderShift	unlimited
**Membrane Region**	
CellOuterBorderShift	-1
CellInnerBorderShift	1
**Object Region**	
ObjectBorderShift	2
**Spot Detection**	
SpotMinimumDistance	2
SpotPeakRadius	0
SpotReferenceRadius	3
SpotMinimumContrast	0.2
SpotMinimumToCellIntensity	0.7

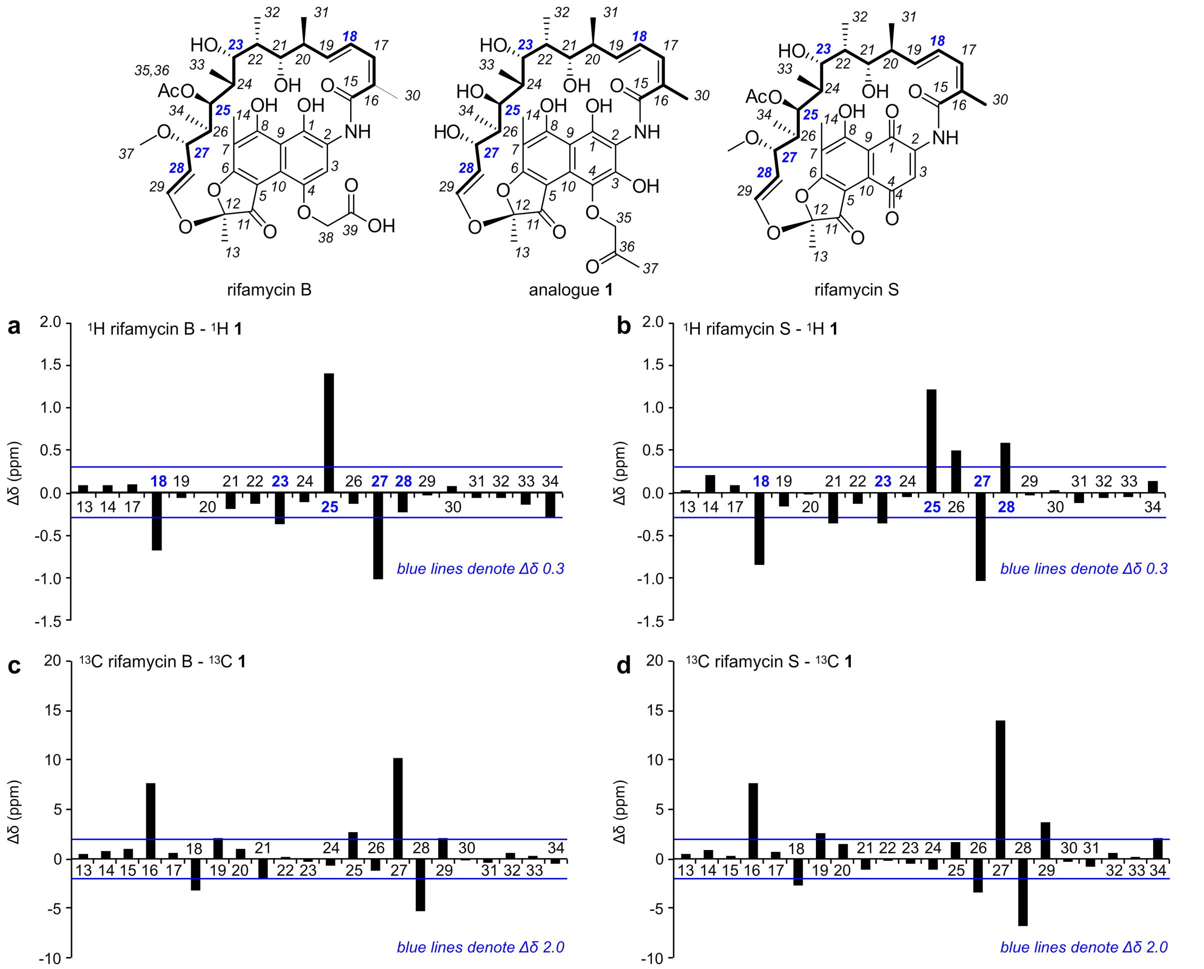

Supporting Figure 1. NMR peak shift analyses. Comparison of the ^{1}H (top row) and ^{13}C NMR (bottom row) peaks of **1** to rifamycin B (left) and rifamycin S (right column). Blue line denotes $\Delta\delta = \pm 0.3$ ppm for ^{1}H and $\Delta\delta = \pm 2.0$ ppm for ^{13}C peaks.

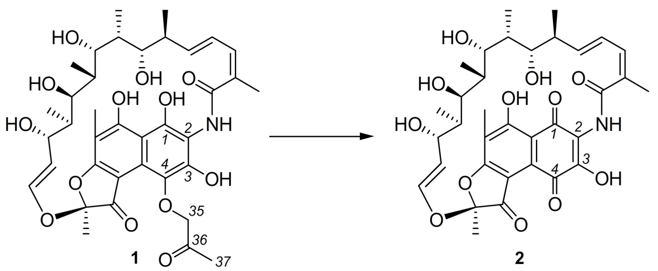

Supporting Figure 2. Proposed degradation of shunt metabolite **1**. Compound **1** was observed to be very reactive. In MeOH solvent it underwent oxidation followed by methanolysis to form **2**. This process is similar to the conversion of rifamycin B to rifamycin S (active form). Traces of **2** (the structure consistent with MS but could not be proven) were always detected when isolating **1** and could not be removed experimentally under the conditions reported herein. Based on prior SAR data, we would anticipate that the activity of this material arises from **2**. Although attempts were made to isolate **2**, the low scale of this effort prevented us from obtaining pure materials.

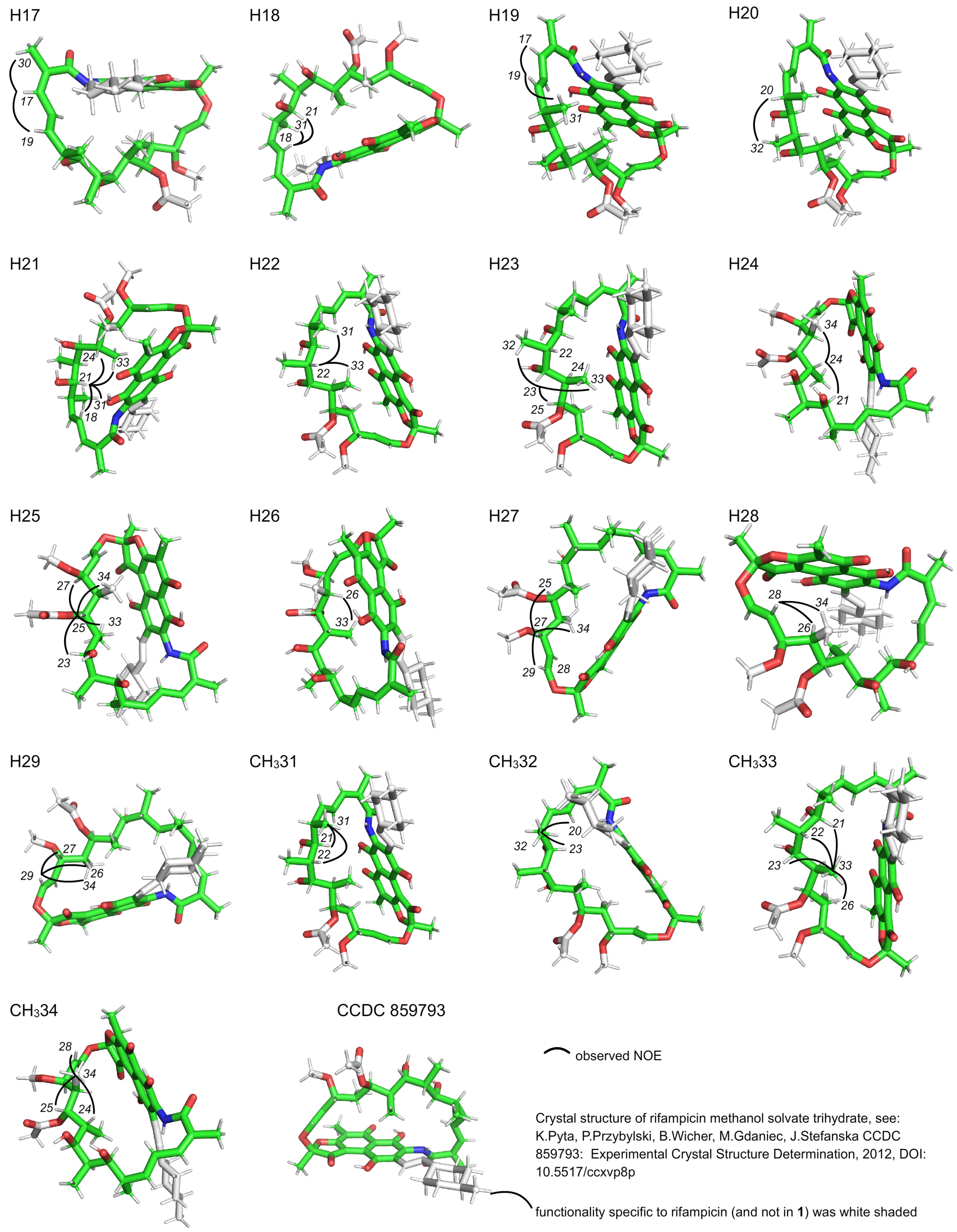

Supporting Figure 3. $^1H,^1H$ NOESY correlations observed for **1** shown on the X-ray crystal structure of rifampicin, indicating the stereochemistry is the same.

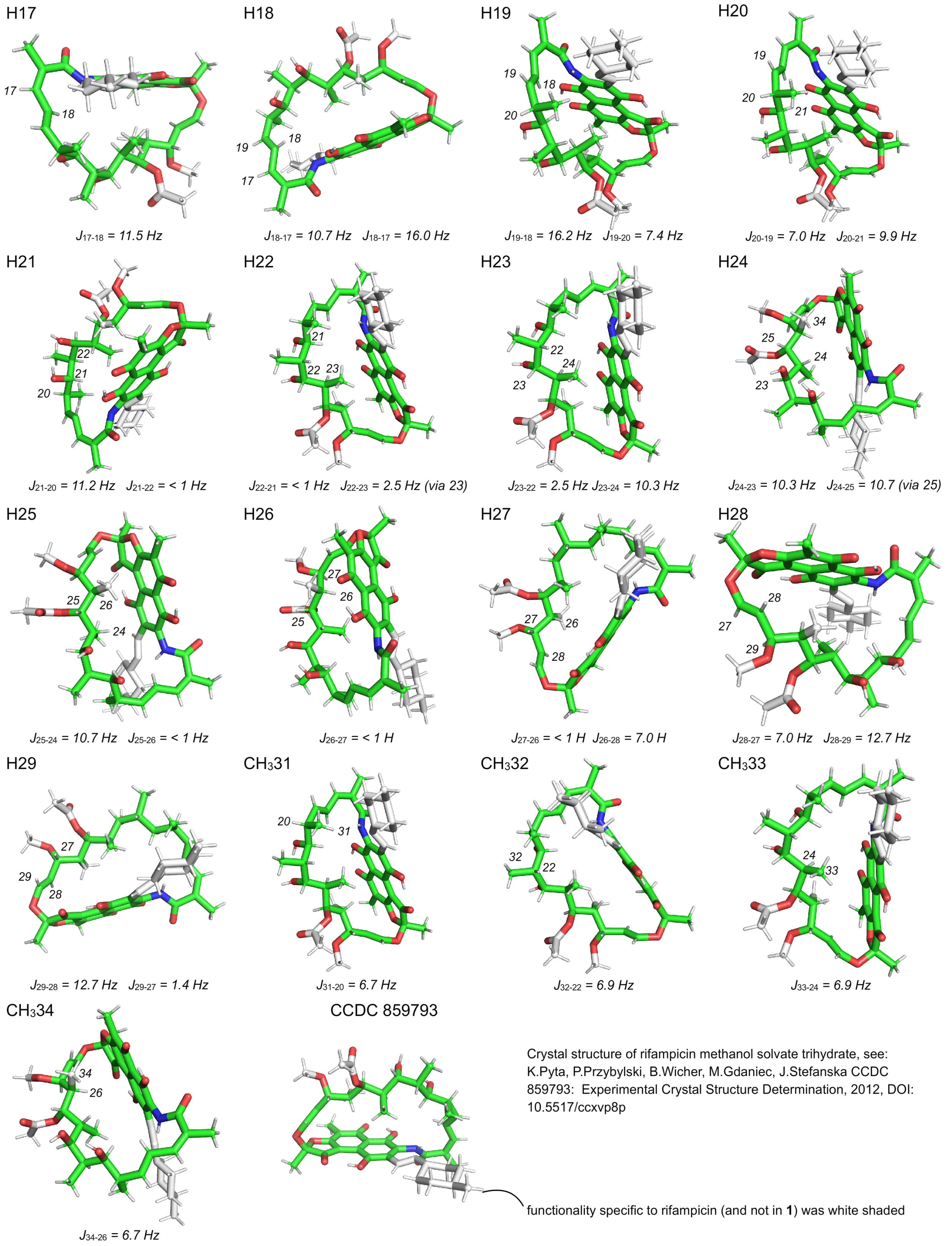

Supporting Figure 4. Coupling constants observed for **1** with the X-ray crystal structure of rifampicin oriented to show that the couplings are consistent with the structure.

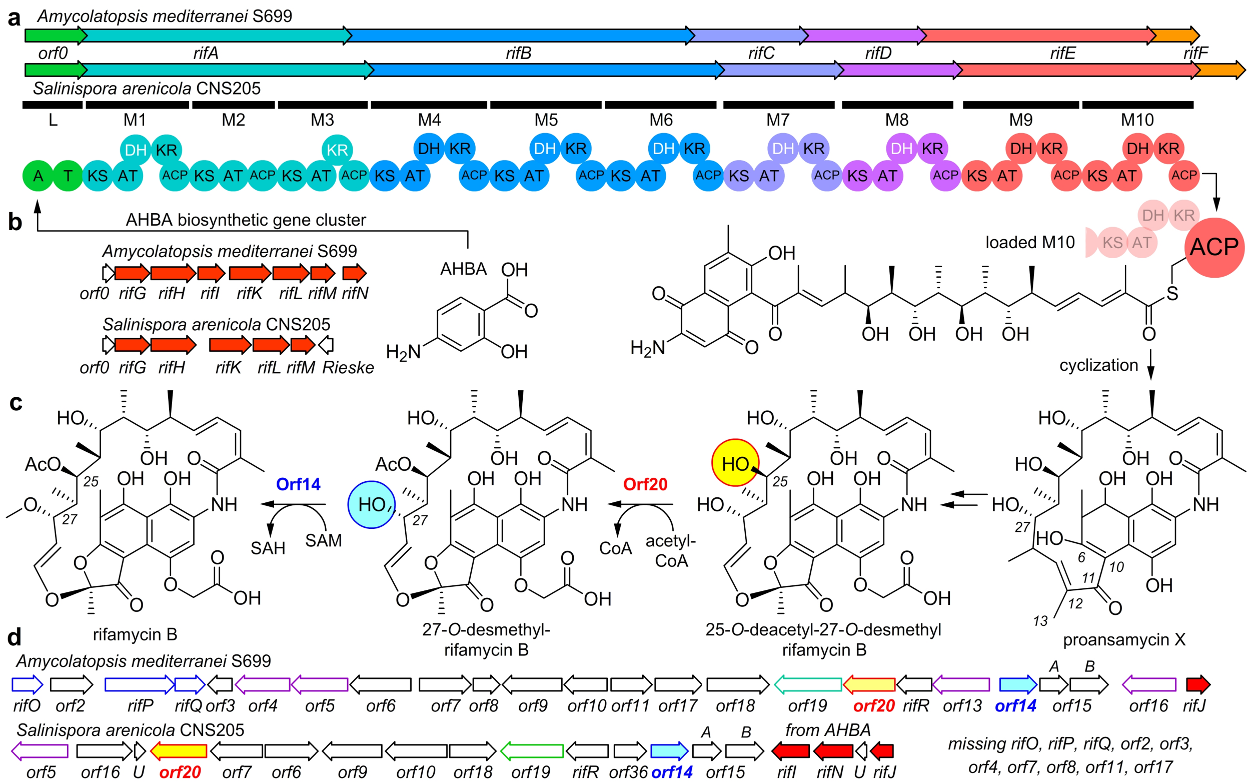

Supporting Figure 5. Comparison of the rifamycin BGCs from *A. mediterranei and S. arenicola.* **a)** The carbon backbone of the rifamycin family is assembled through a polyketide synthase (PKS). PKS assembly involves *orf0* and *rifA-rifF* with modular assemblies comprised of AT (acyltransferase), ACP (acyl carrier protein), DH (dehydratase), and KR (ketoreductase) domains. PKS assembly ends at *rifE* where the substrate is released from the terminal ACP in module 10 through amide-forming macrocyclization to deliver proansamysin X. Both strains share very similar PKSs. **b)** The PKS pathway begins with a 3-amino-5-hydroxy benzoic acid (AHBA) starting unit. In *A. mediterranei*, 8 genes (*orf0-rifN*) are located within one cluster, while *rifJ* is located within a region that contains the post-PKS tailoring enzymes. In *S. arenicola*, *rifI* and *rifN* appear proximal to *rifJ*. Amino acid similarities of ~75% are observed in the PKS and AHBA genes between *A. mediterranei* and *S. arenicola*. **c**) Conversion to rifamycin SV arises through a multistep process that begins with elaboration of the 5-membered ring between C6 and C12 to form 25-*O*-deacetyl-27-*O*-desmethyl rifamycin B, which undergoes a two-step acetylation and methylation sequence to achieve rifamycin B through CoA-guided acylation by orf20 and SAM-dependent methylation by orf14. **d)** Noticeably different post-PKS rifamycin modification enzymes are observed in *A. mediterranei* and *S. arenicola,* as shown by this gene comparison.

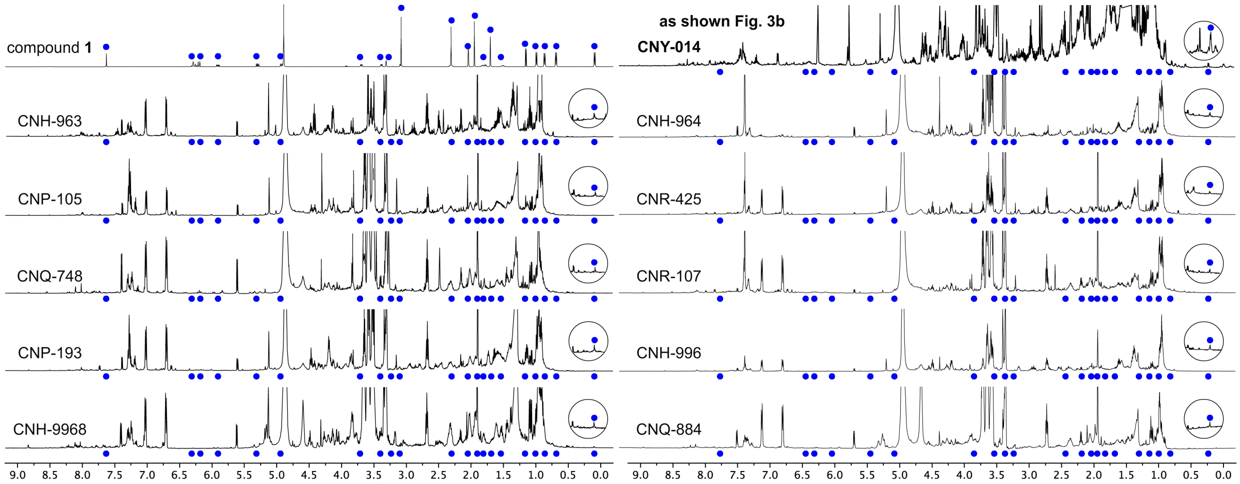

Supporting Figure 6. ^{1}H NMR spectrum of compound **1** (top row, peaks marked with blue dots) as compared to the crude extracts of ten additional strains of *S. arenicola*. All the extracts show the presence of compound **1**.

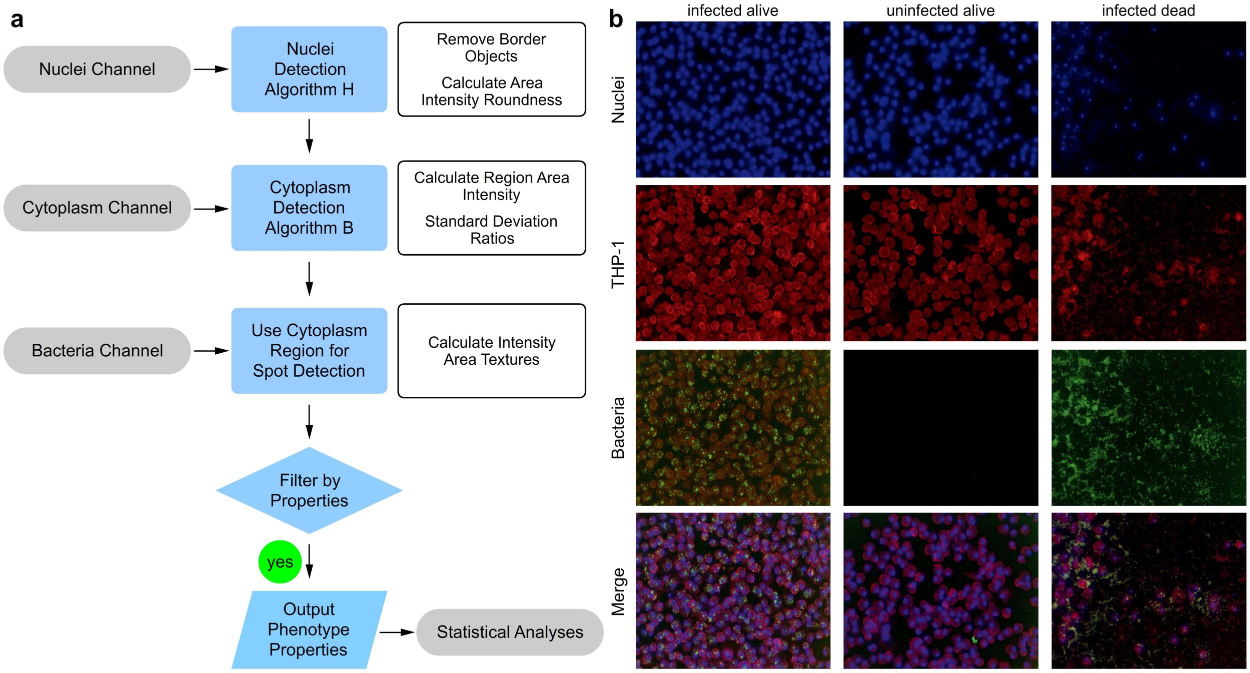

Supporting Figure 7. Acapella image analysis. **a)** Flowchart describing sequential steps used in image analysis. **b)** Example images visualising *S.aureus* infected THP-1 cells with positive control (left column) and negative control (right). For comparison, uninfected THP-1 cells in media only are shown in the middle column.

^{1}H-NMR (600 MHz) spectra of **1** in CD_3OD

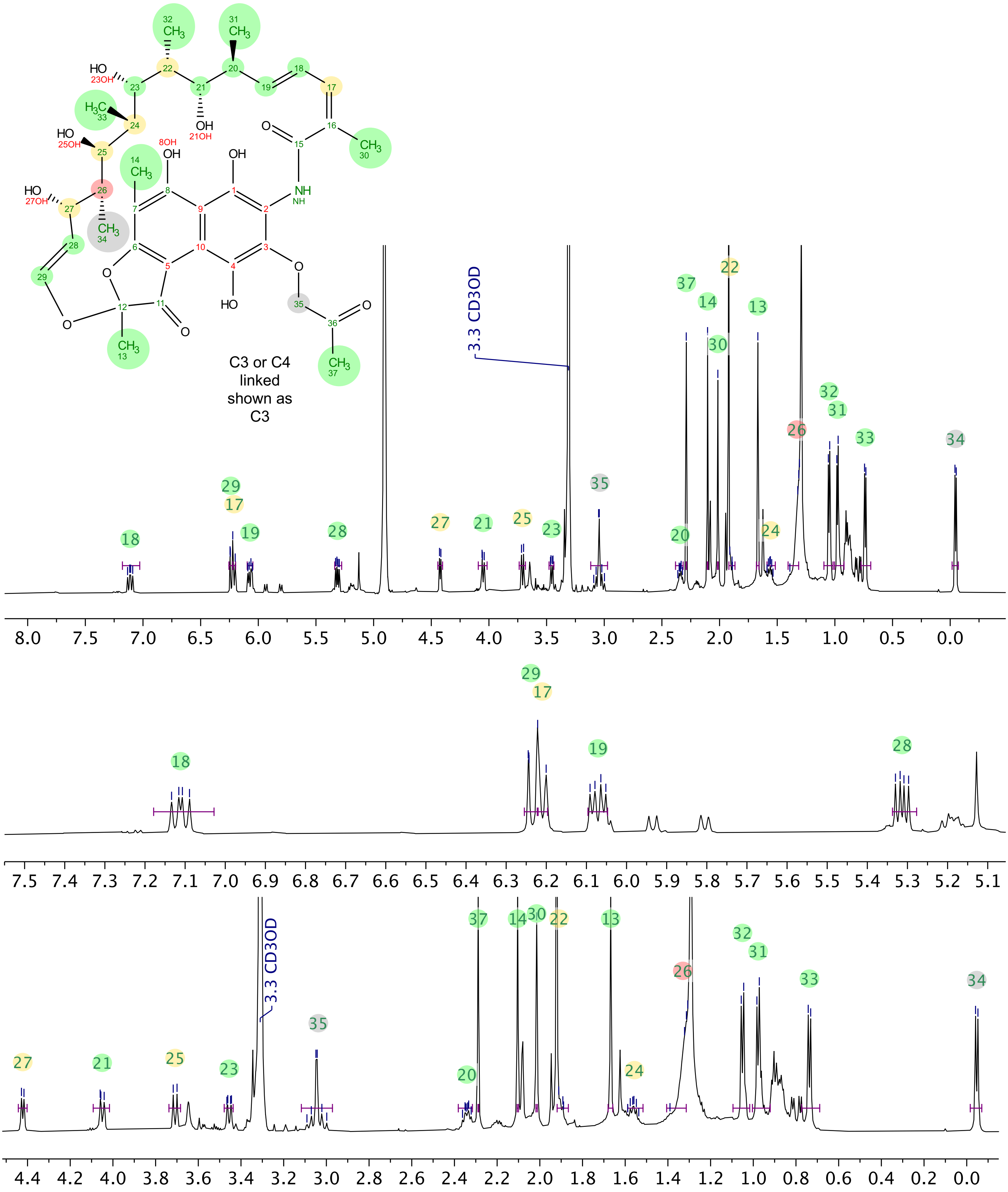

$^{1}H,^{1}H$-gCOSY (600 MHz) spectrum of **1** in CD_3OD

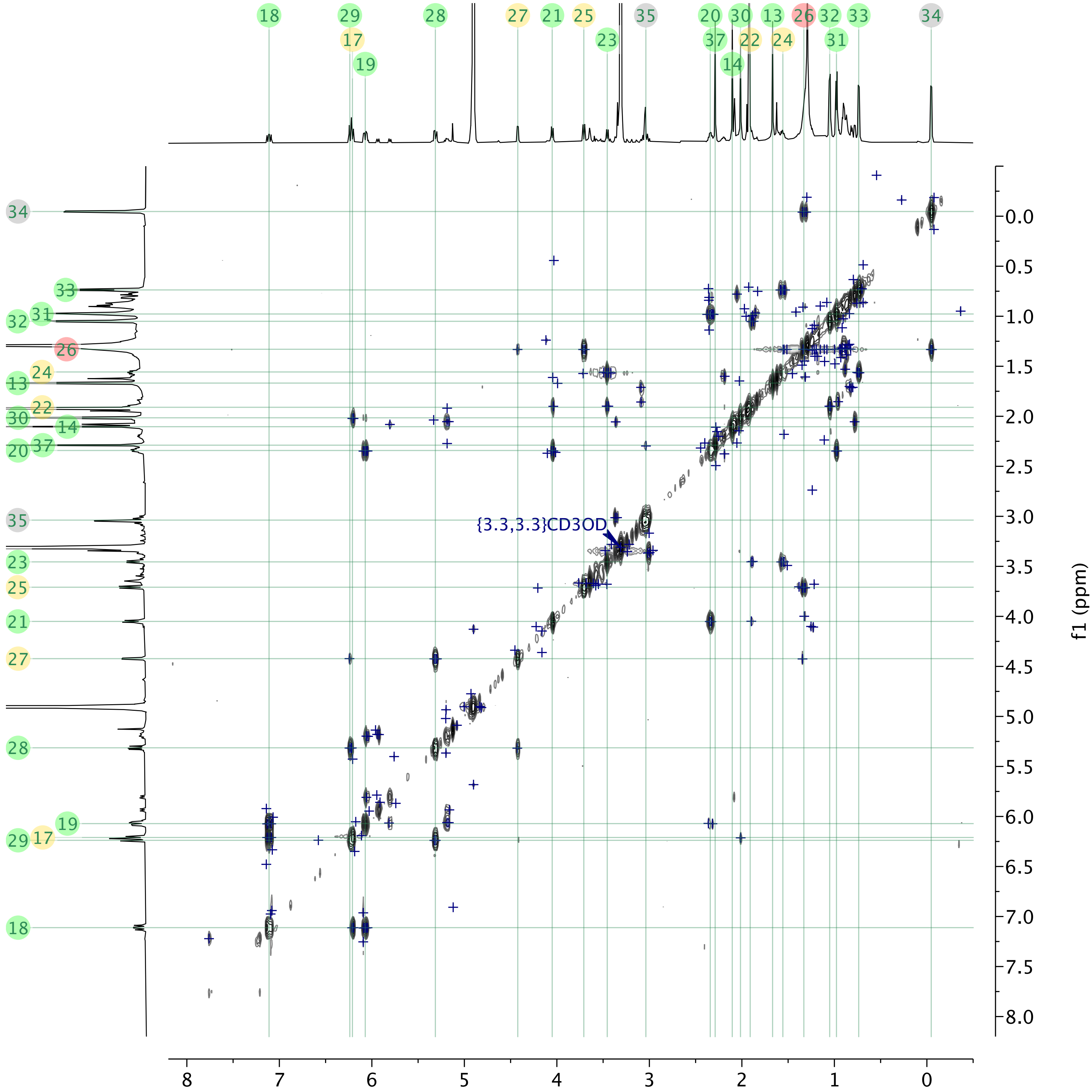

$^{1}H,^{1}H$-NOESY (600 MHz) spectrum of **1** in CD_3OD

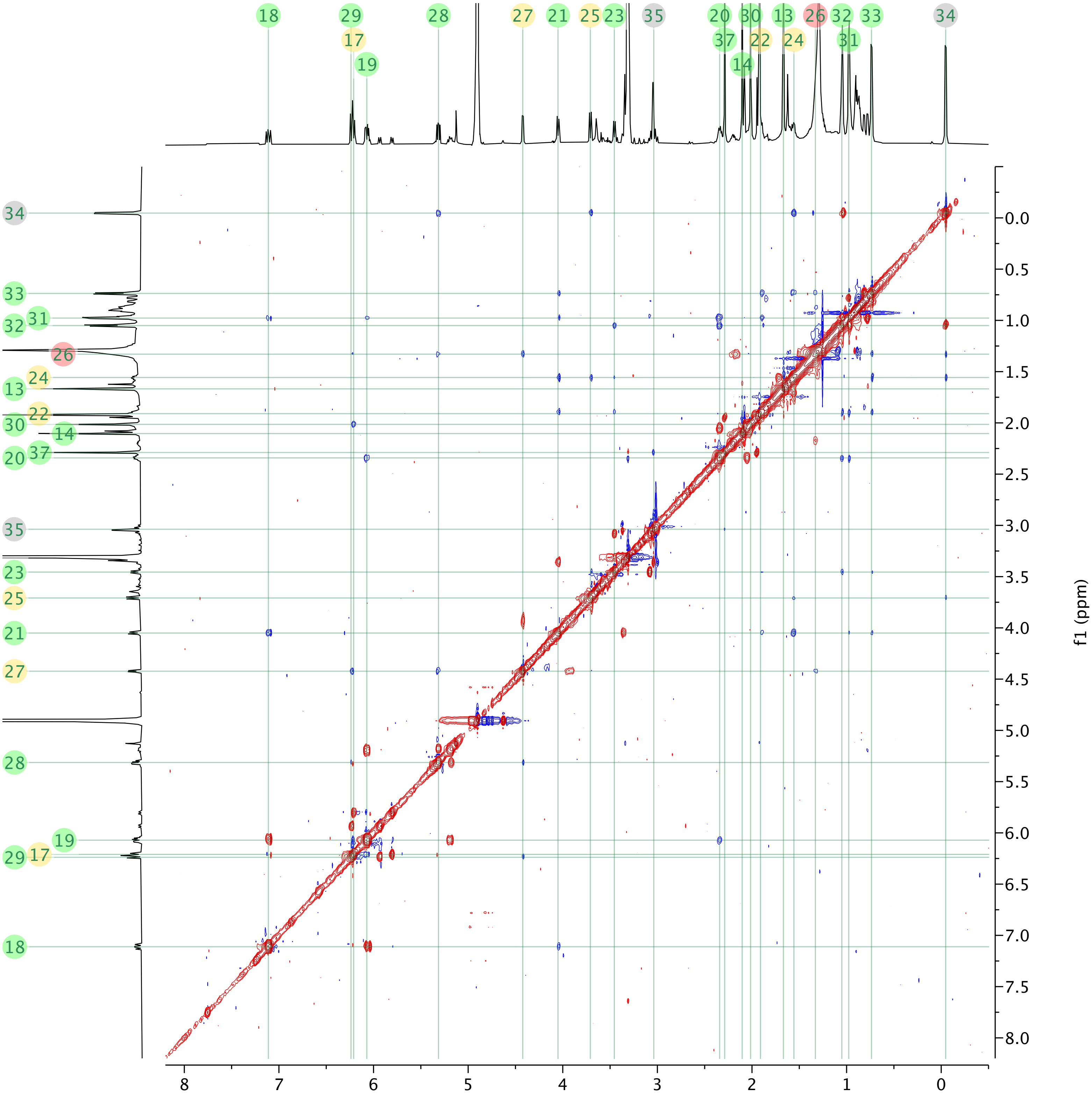

$^1H,^{13}C$-HSQC (600 MHz) spectrum of **1** in CD_3OD

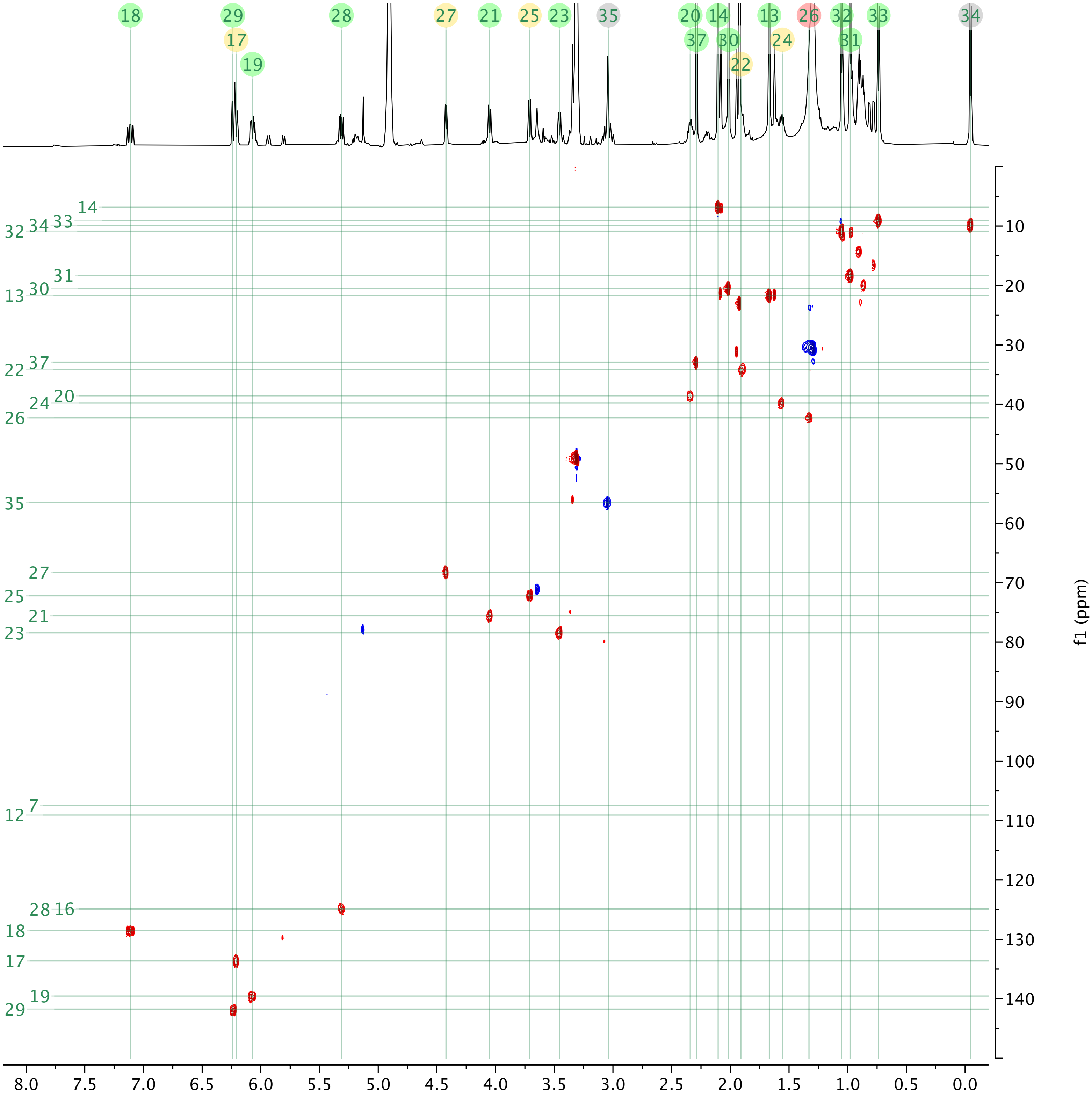

$^{1}H,^{13}C$-HMBC (600 MHz) spectrum of **1** in CD_3OD

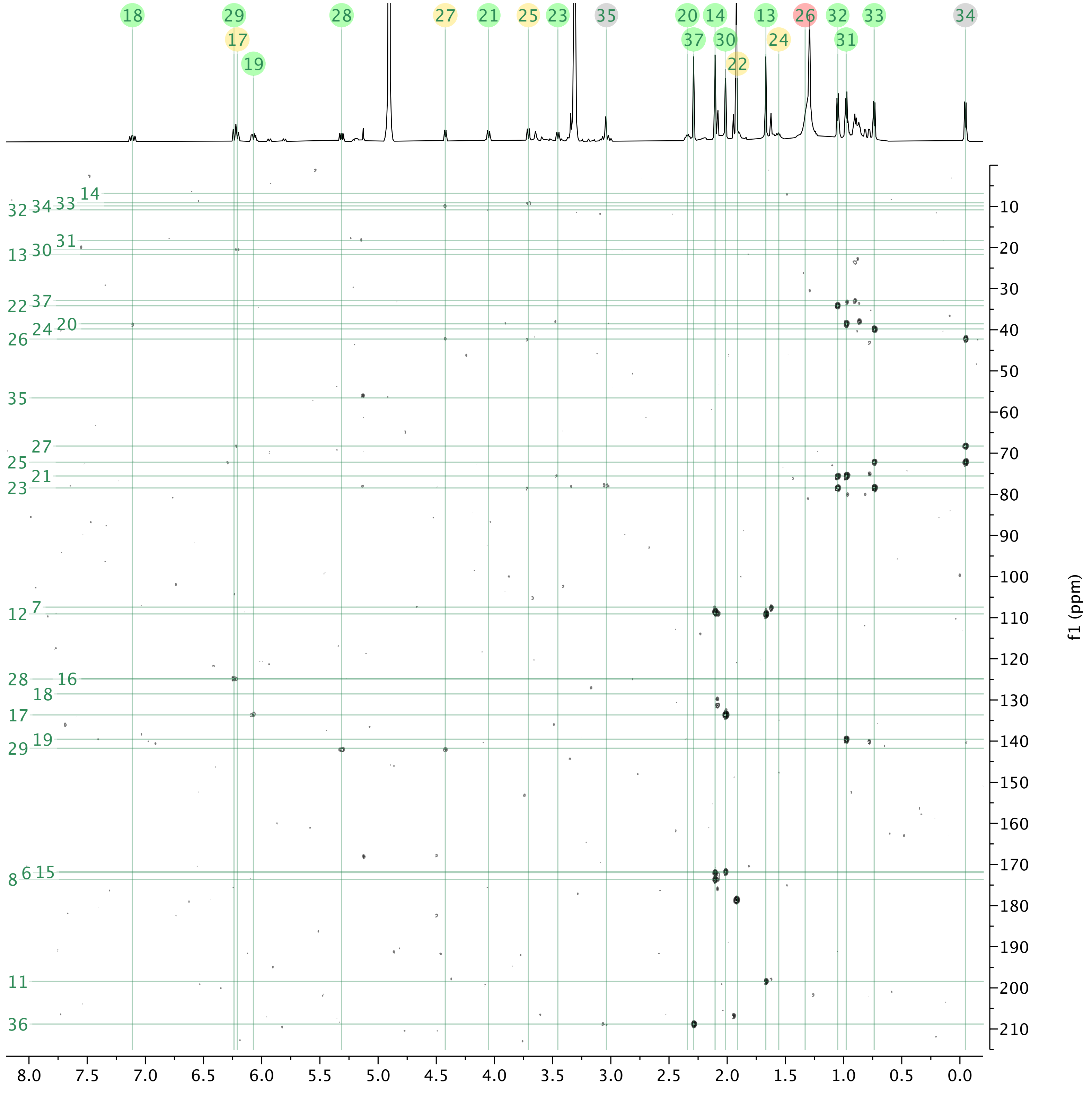

^{1}H-NMR (600 MHz) spectra of rifamycin B in CD_3OD

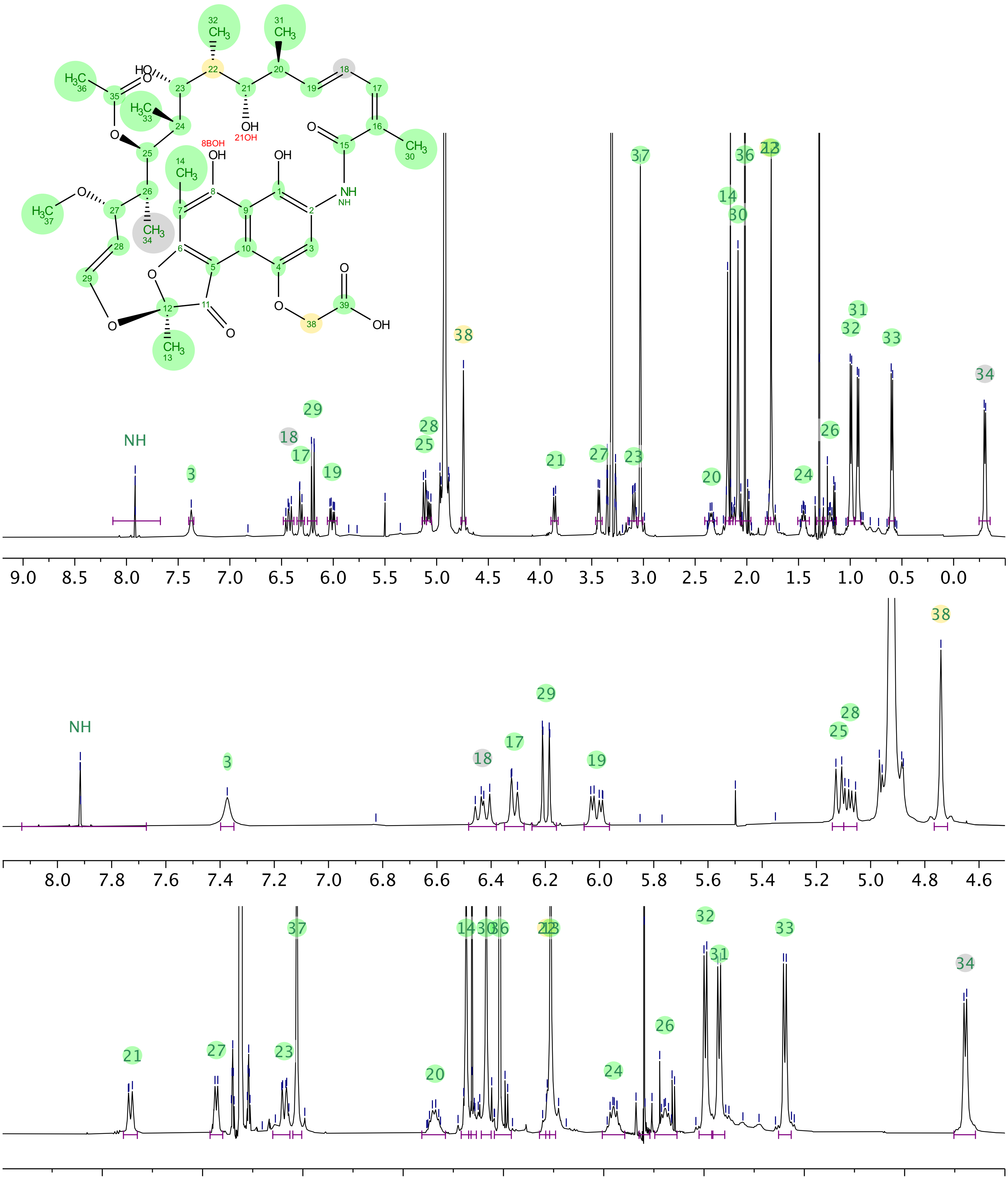

^{13}C-NMR (125 MHz) spectra of rifamycin B in CD_3OD

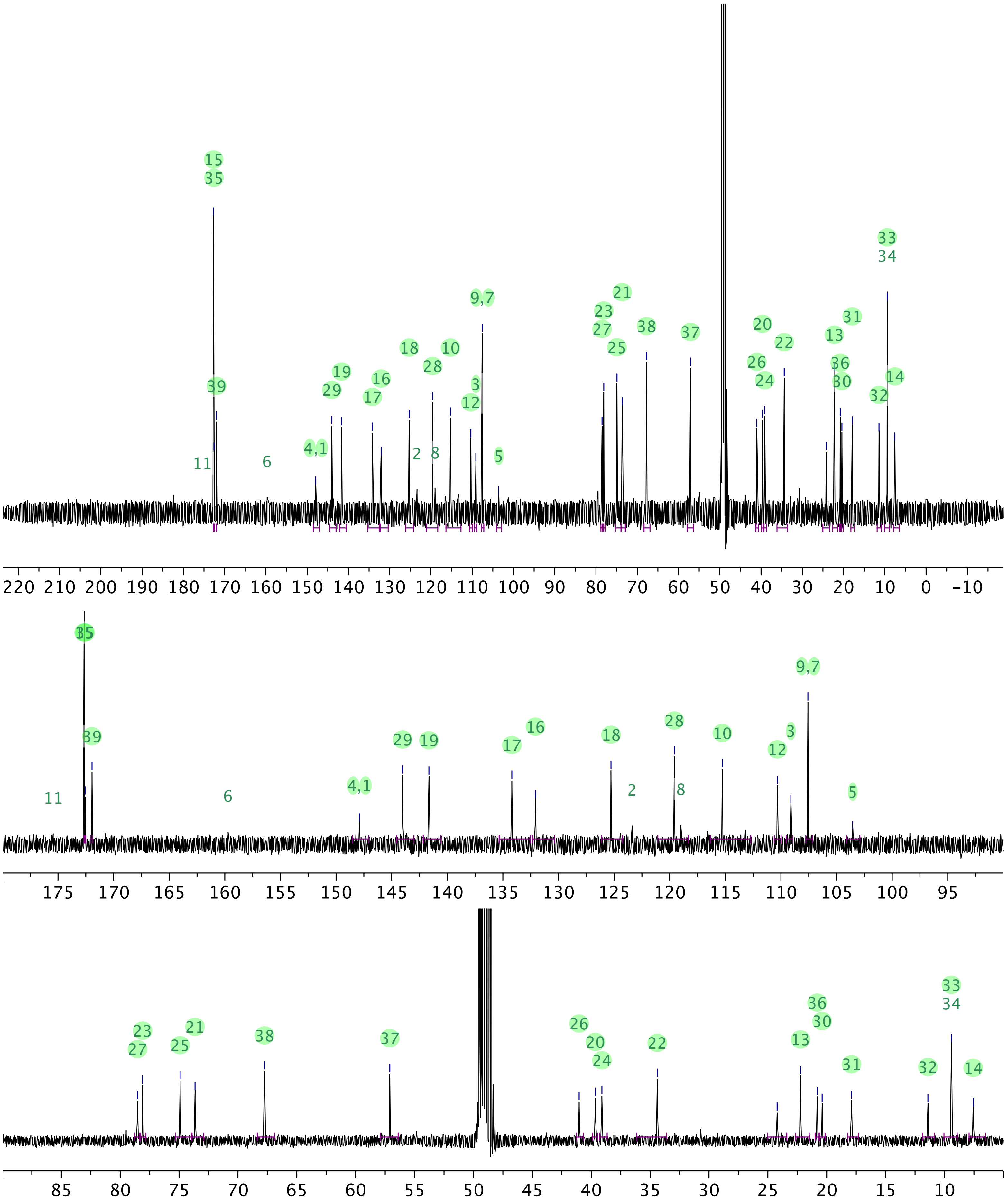

$^1H,^1H$-gCOSY (600 MHz) spectrum of rifamycin B in CD_3OD

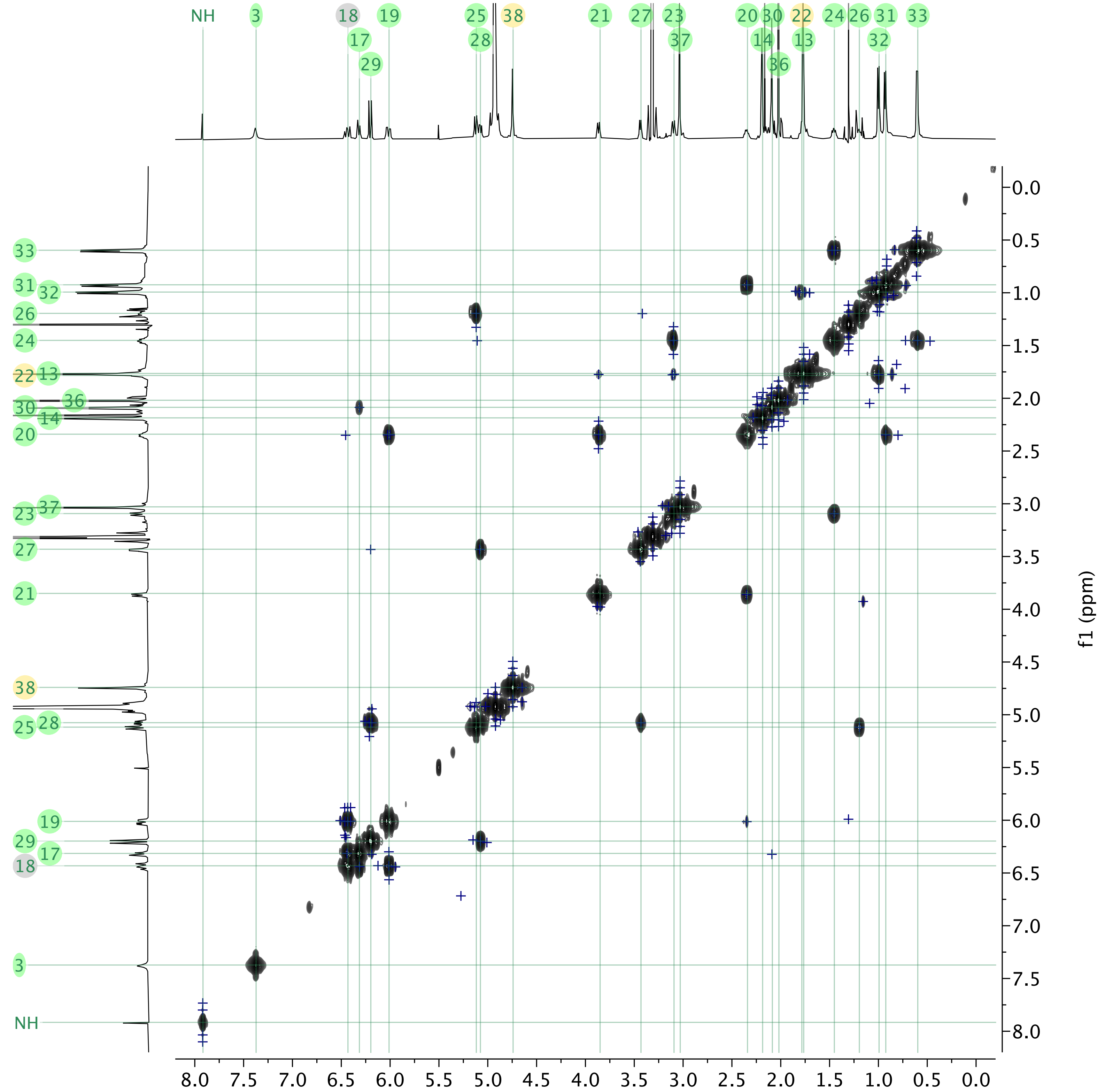

$^{1}H,^{13}C$-HSQC (600 MHz) spectrum of rifamycin B in CD_3OD

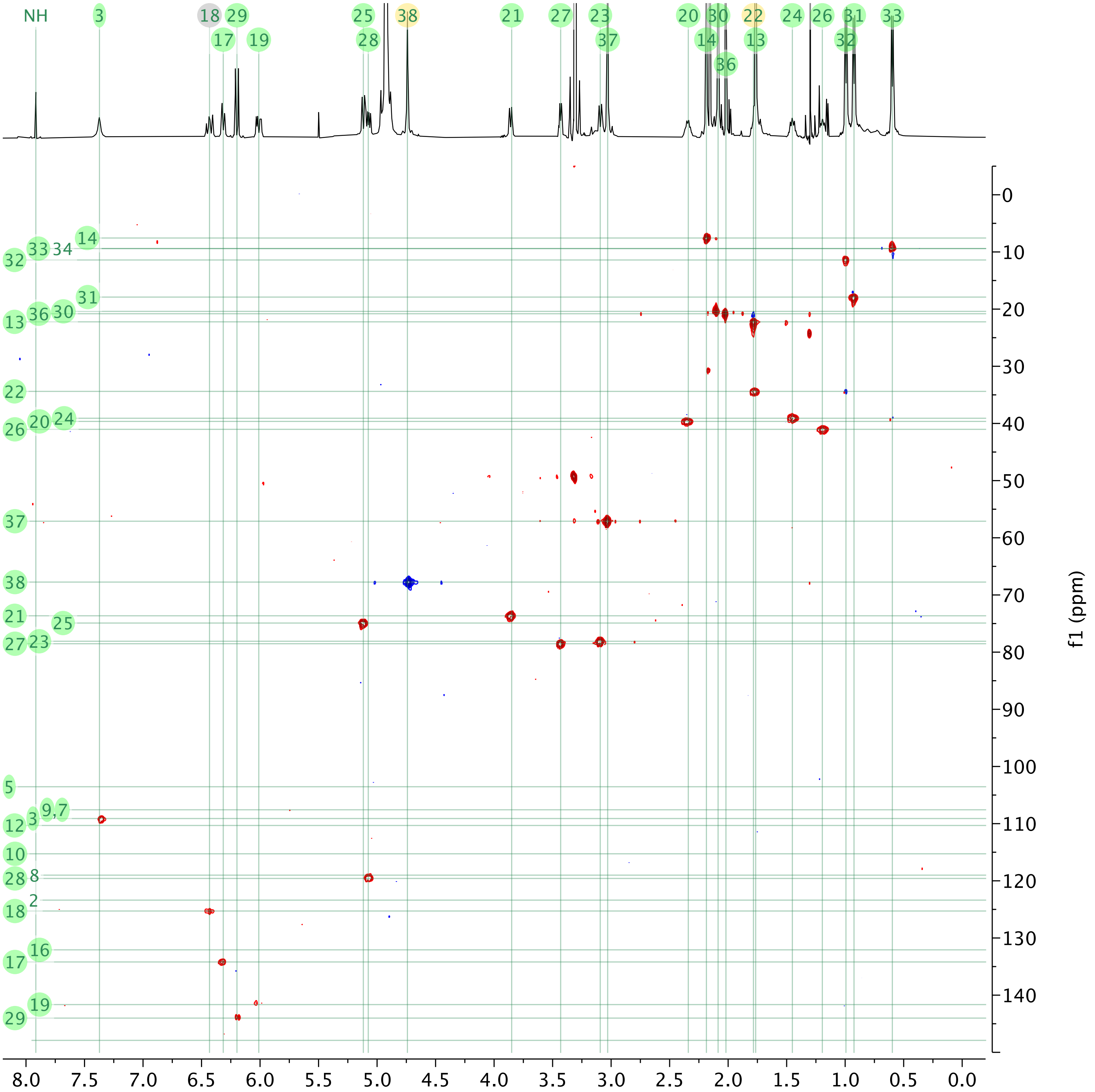

$^{1}H,^{13}C$-HMBC (600 MHz) spectrum of rifamycin B in CD_3OD

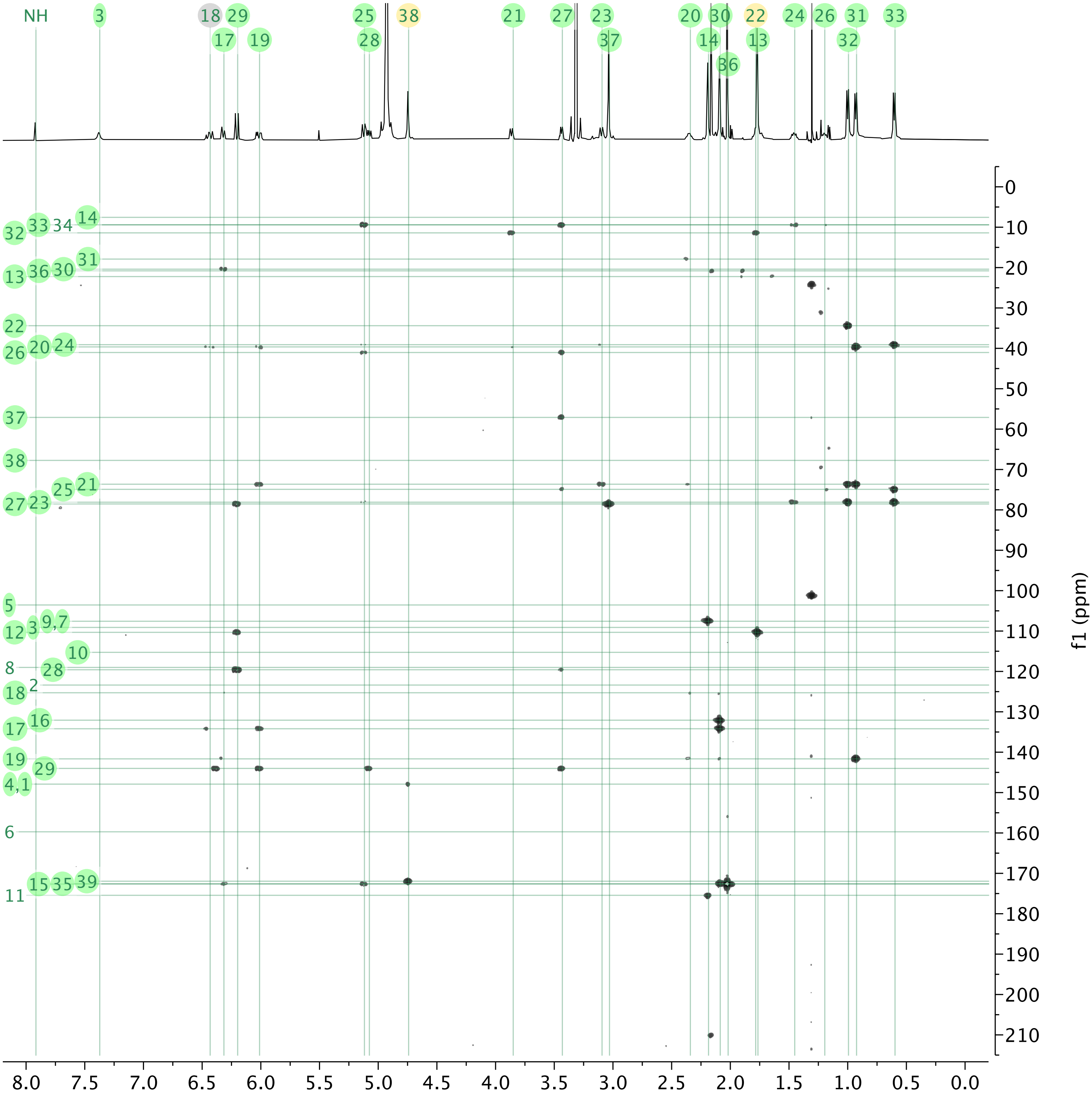

^{1}H-NMR (600 MHz) spectra of rifamycin S in CD_3OD

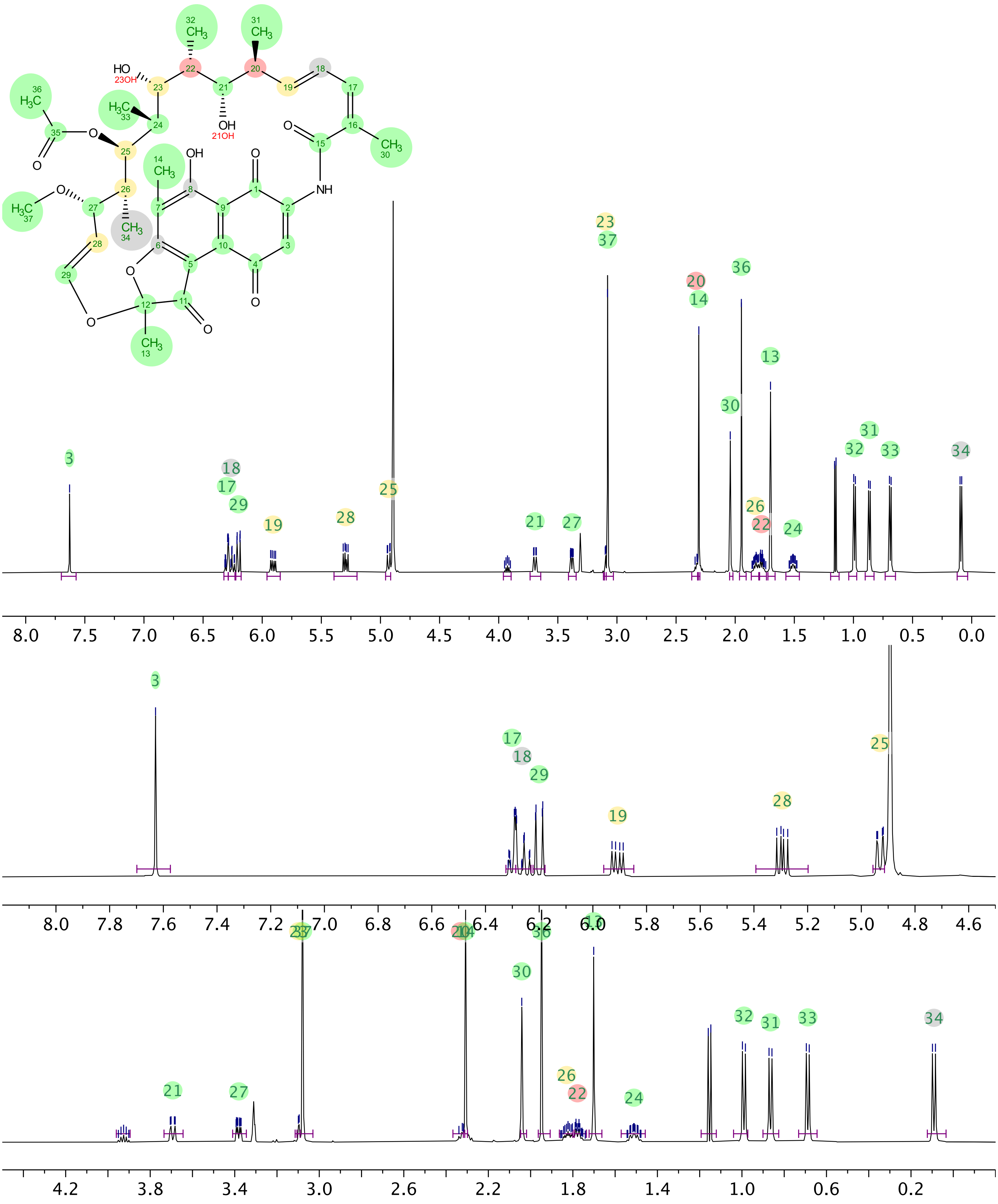

^{13}C-NMR (125 MHz) spectra of rifamycin S in CD_3OD

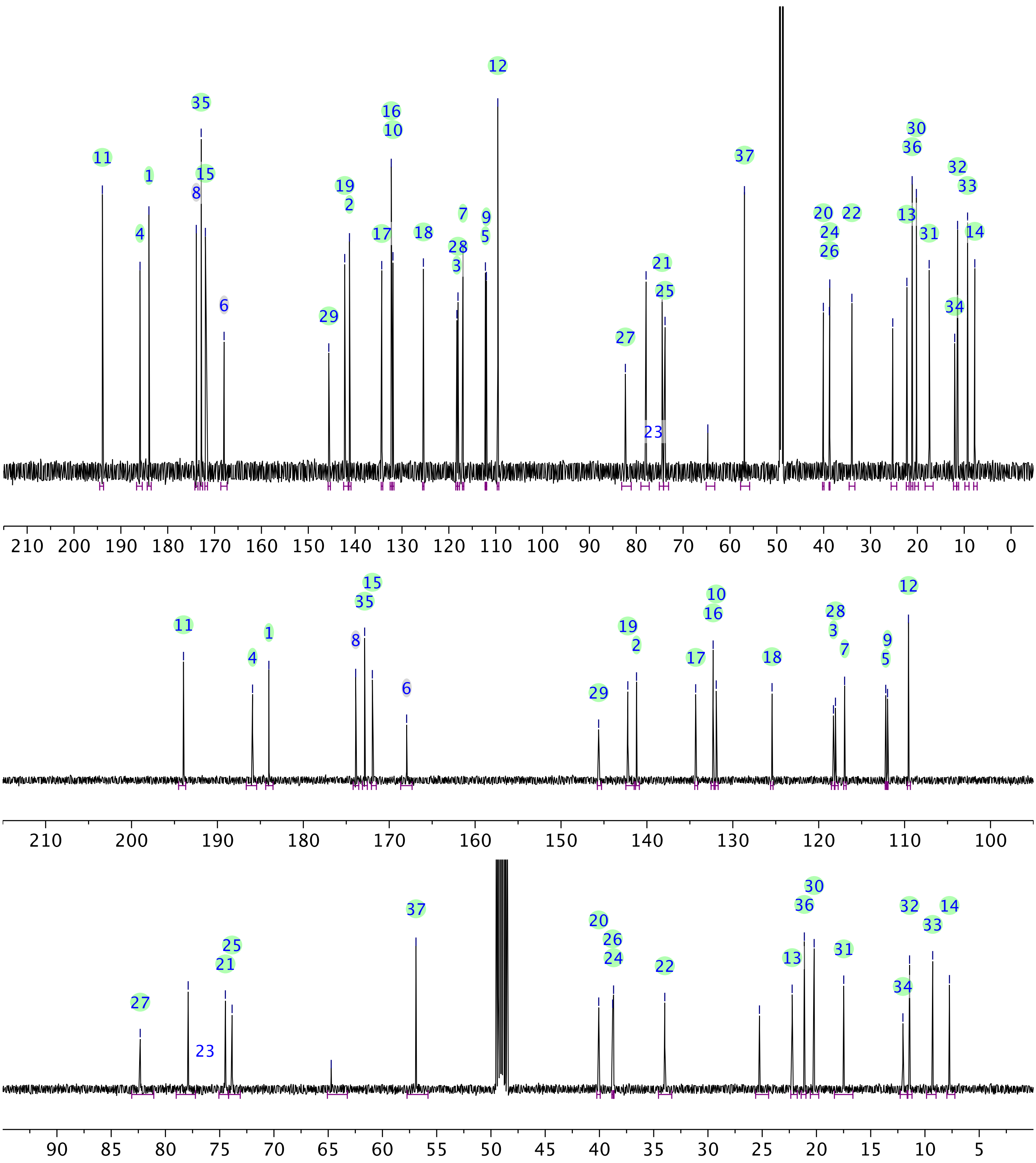

$^{1}H,^{1}H$-gCOSY (600 MHz) spectrum of rifamycin S in CD_3OD

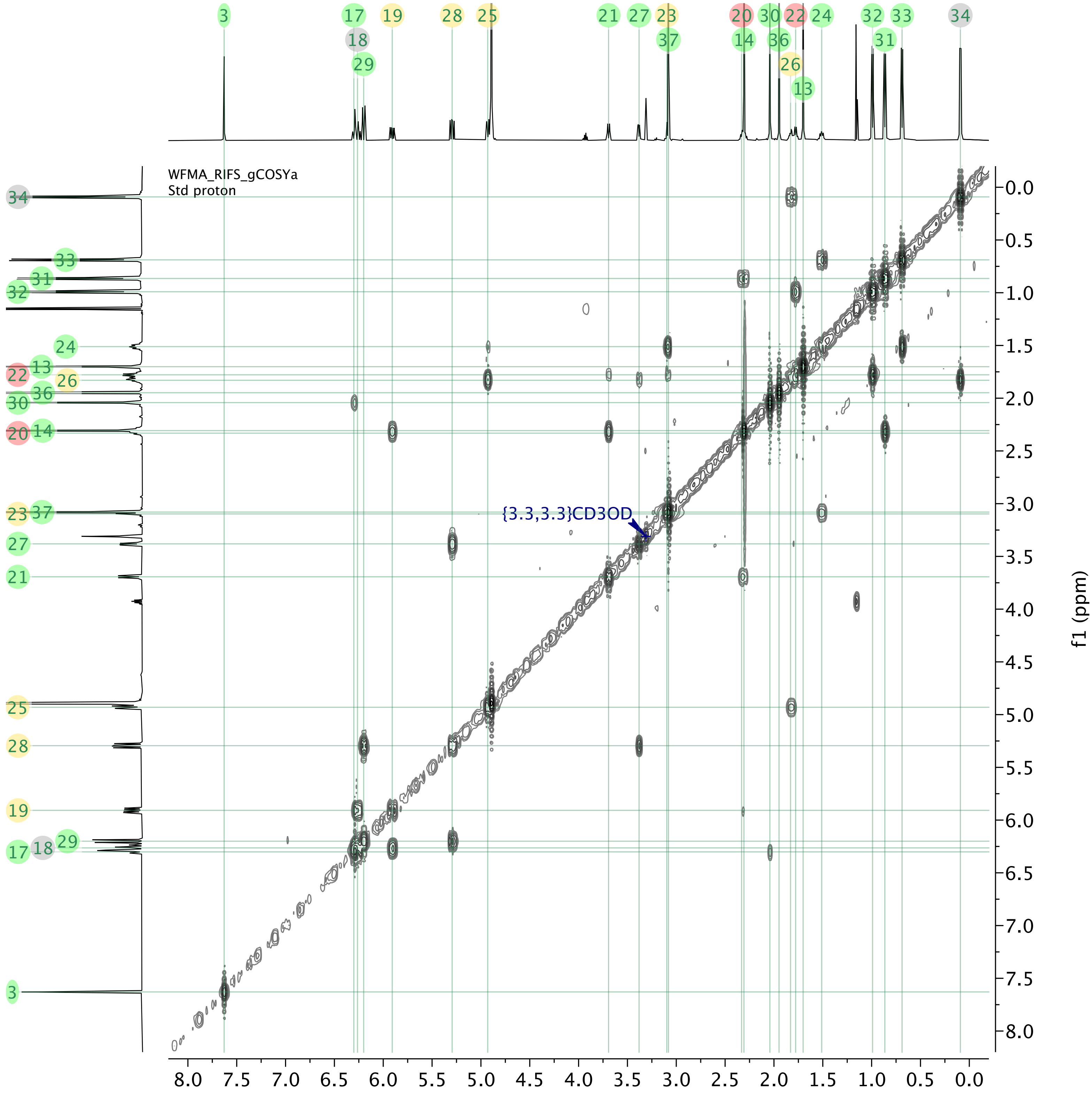

$^{1}H,^{13}C$-HSQC (600 MHz) spectrum of rifamycin S in CD_3OD

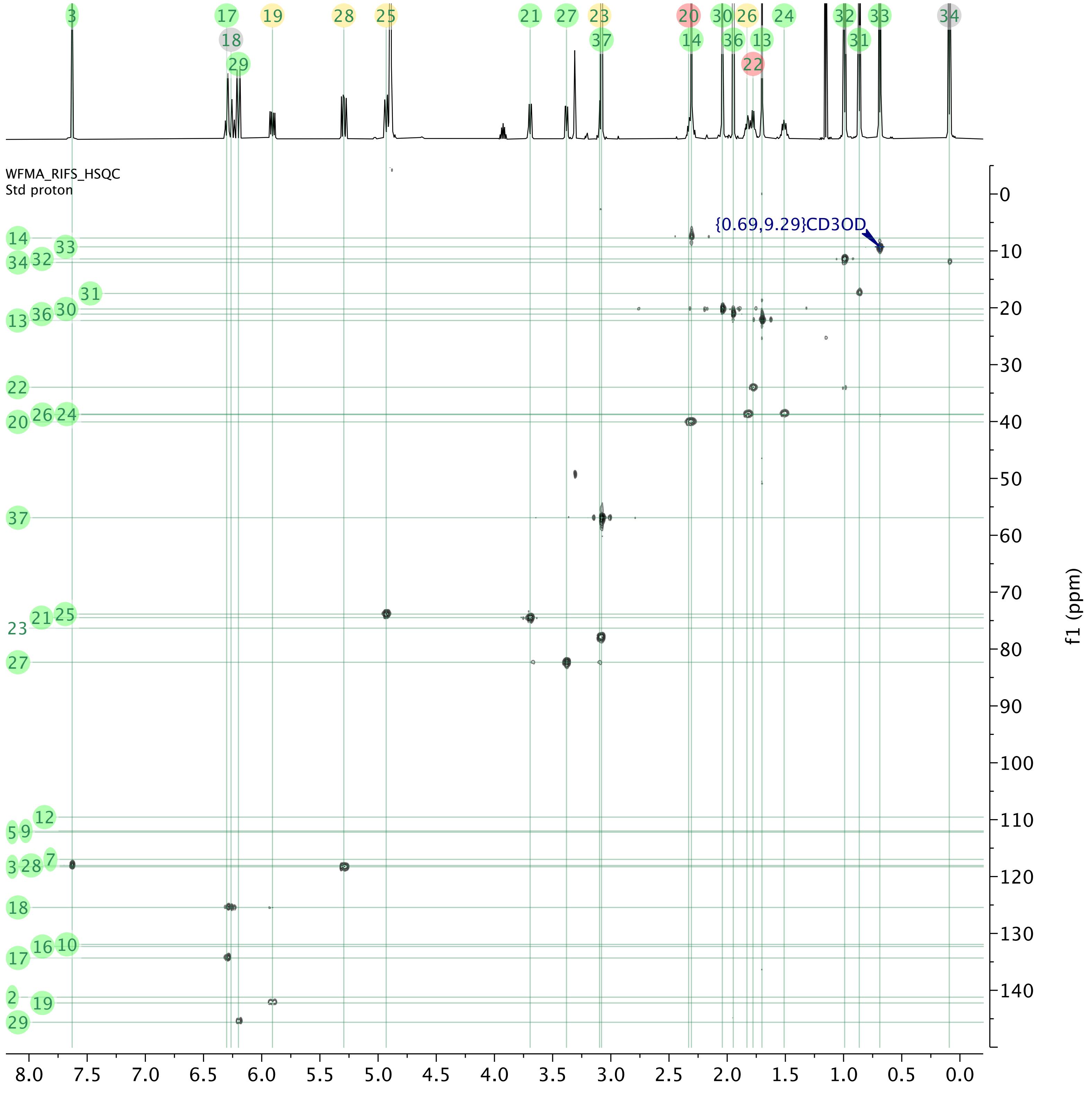

$^{1}H,^{13}C$-HMBC (600 MHz) spectrum of rifamycin S in CD_3OD

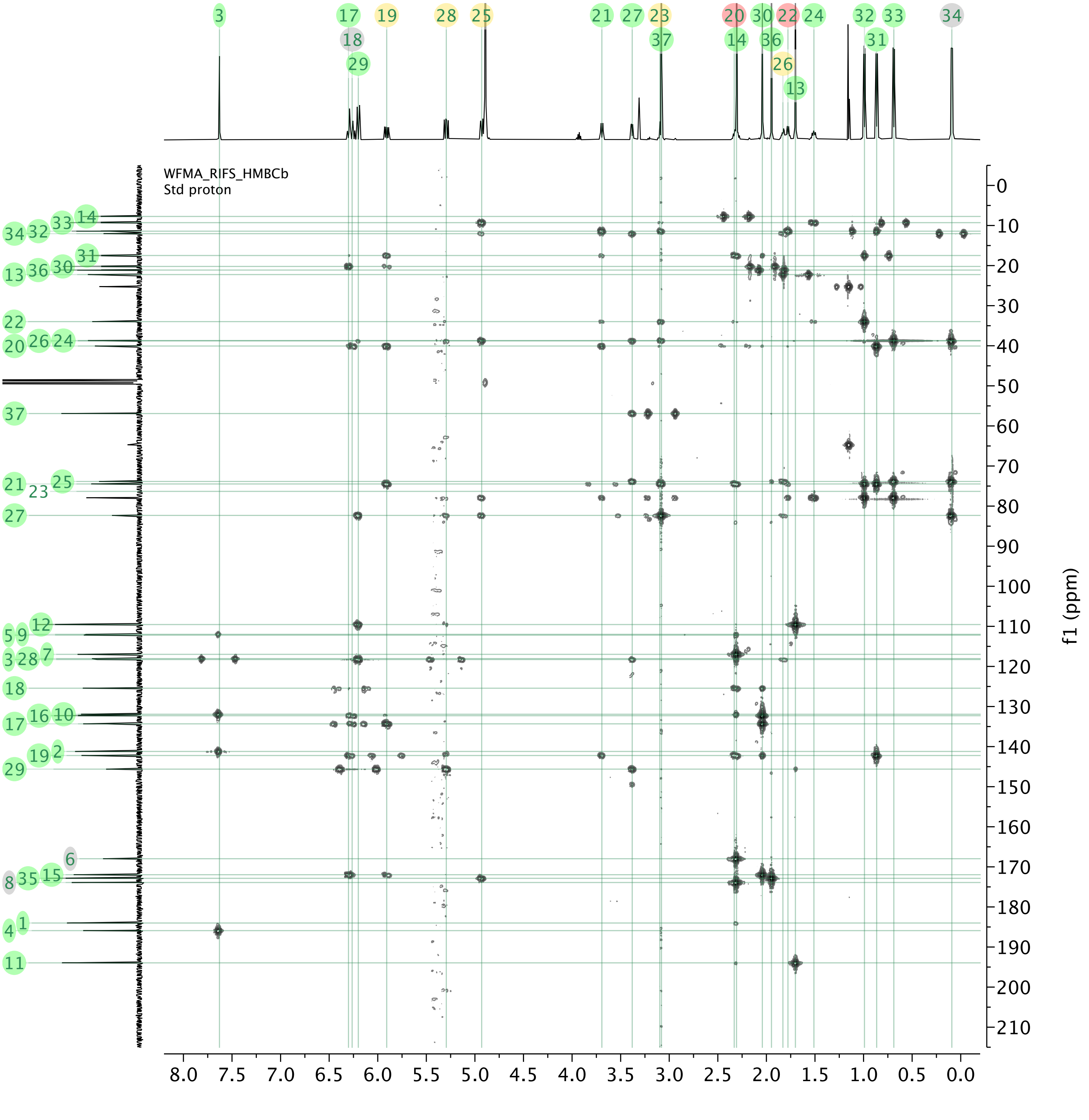

HRMS spectrum of **1**

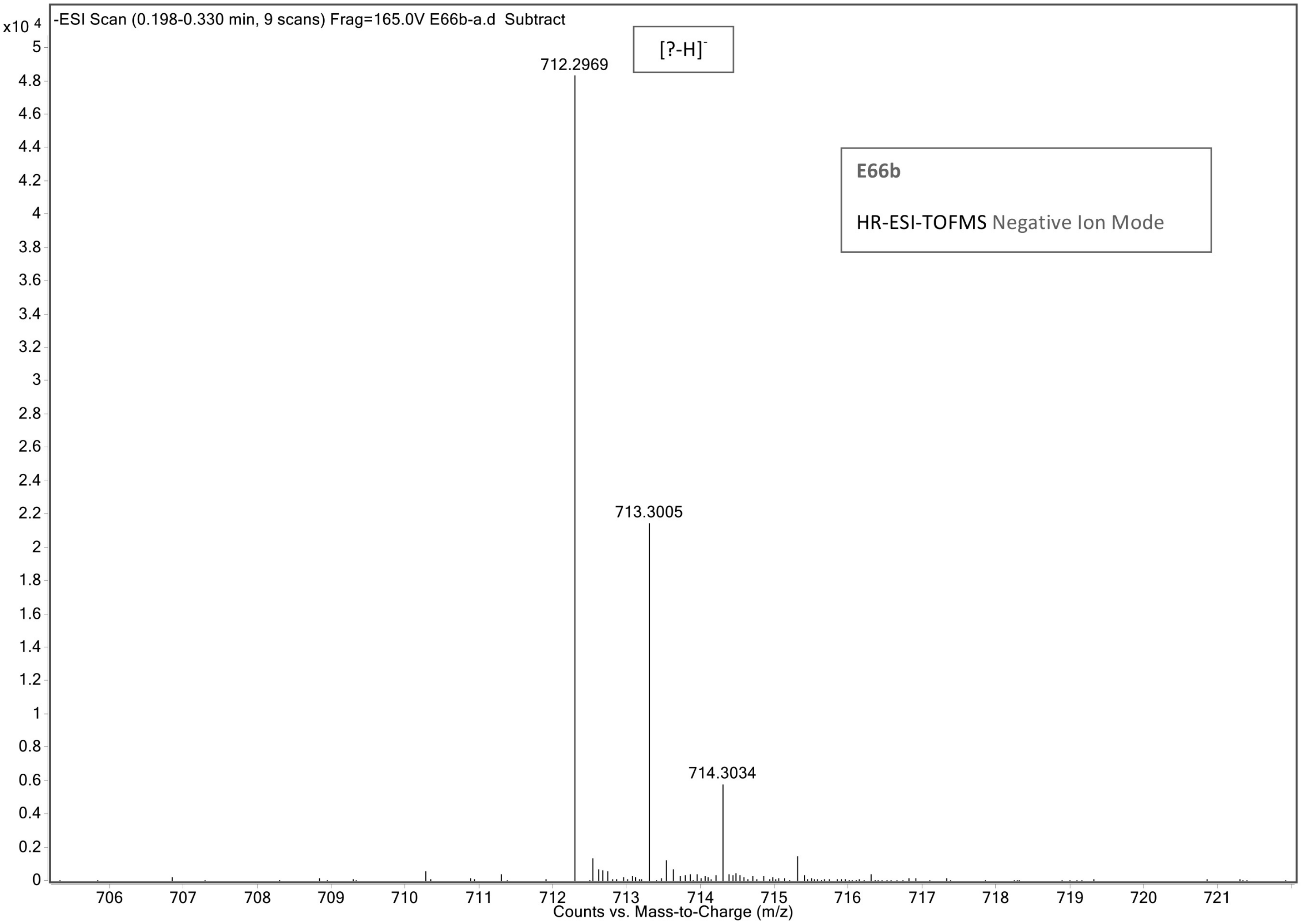

Supplement: Supplementary file 1 — id3c00049_si_001.pdf [file id3c00049_si_001.pdf]
